# Hybrid Plasmonic Photoanodes for Effective Photocatalytic Water Splitting

**DOI:** 10.1002/nap2.70237

**Published:** 2026-08-13

**Authors:** Hongkai Zhou, Andrew Campbell, Wilfrid Harvey, Vasyl G. Kravets, Alexander N. Grigorenko

**Affiliations:** ^1^ Department of Physics and Astronomy the University of Manchester Manchester UK

**Keywords:** aluminium diboride, green hydrogen, metamaterial photoanodes, plasmonic photocatalyst, transition metal dichalcogenides, water splitting

## Abstract

Photocatalytic water splitting by semiconductors is elucidated in many important works. At the same time, mechanisms of plasmonic enhancement of photocatalytic water splitting by metals are still debated. Here, we study hybrid photocatalysts in which a main metallic catalyst, AlB_2_, is combined with a co‐catalyst made of either a metal, a semiconductor or a dielectric (FeSe, WS_2_ and ZrO_2_). These catalysts are fabricated from AlB_2_ and relevant powders by a low‐cost, high‐yield and scalable rolling mill technique. They demonstrate photocatalytic water/seawater splitting under solar‐like radiation with high efficiency. We show that the reasons for efficiency enhancement are connected to optical impedance matching in hybrid photoanodes, reasonable light absorption that allowed total light absorption in 300 nm films, and matching the absorption spectrum of photoanodes to the spectrum of solar light. Using hybrid photoanodes composed of AlB_2_ and FeSe nanostructures, we achieved a maximum applied bias photon‐to‐current efficiency of ∼10% under simulated solar illumination with an applied bias of 0.3 V, which brings our approach within values for the commercial viability benchmark. We also demonstrate seawater splitting using stable AlB_2_‐FeSe and AlB_2_‐WS_2_ photoanodes attaining current densities of ∼5 mA/cm^2^ by solar illumination raising efficiency of seawater splitting to ∼5%. Bifunctionality of studied hybrid photoanodes is established.

## Graphical abstract

Photocatalytic water and seawater splitting with high efficiency (∼10%) by hybrid plasmonic photoanodes based on a metamaterial made of metal nanostructures.

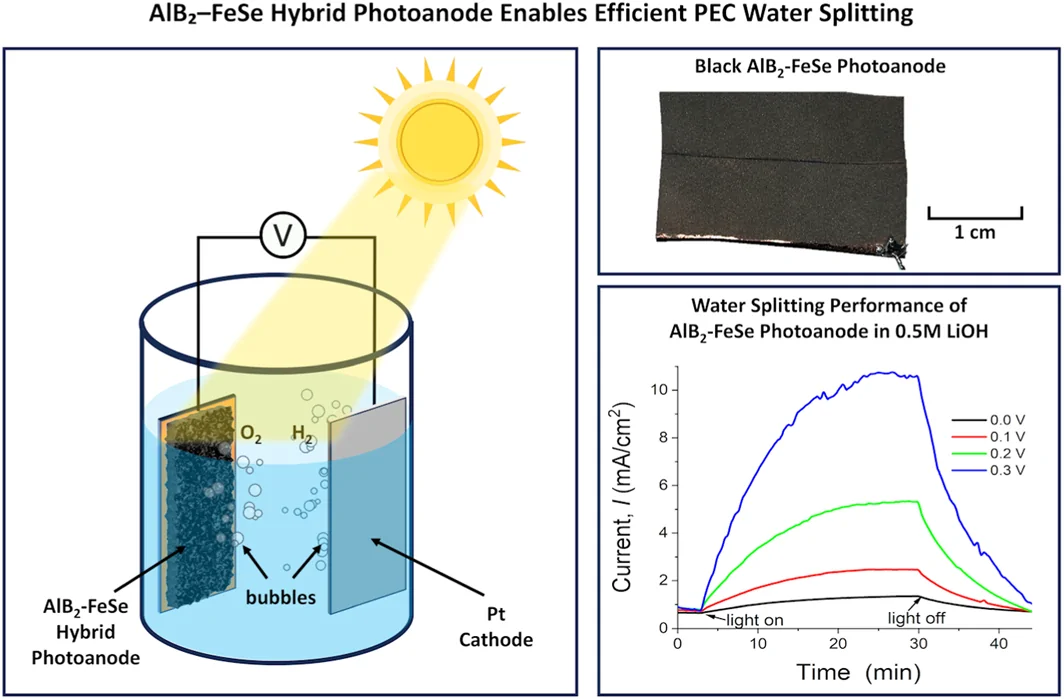

## 1 Introduction

In forthcoming future, green hydrogen could become an important player in the energy market. Among currently available hydrogen (H_2_) production techniques, photoelectrochemical (PEC) water splitting has one of the lowest environmental impacts. Historically, PEC water splitting has received much attention as a promising technology for solar hydrogen production after Fujishima and Honda demonstrated it using a TiO_2_ photoanode in 1972 [1]. The performance of a PEC cell is mainly determined by its photoanode, which (ideally) should be made of earth‐abundant materials that are stable in the water and offer an applied bias photon‐to‐current efficiency (ABPE) above 10% to transform the PEC cell into a commercially viable solar H_2_ production device [2, 3].

Nowadays, despite extensive research worldwide, the ABPE efficiencies of studied photoanodes (mostly made of semiconductors and dielectrics) are still far below the 10% target simply because their large energy bandgaps and charge transport properties are not appropriate for efficient sunlight harvesting and conversion [4, 5]. It is worth stressing that there is no reports of photoanode technology that would meet the following three important requirements: (i) ABPE conversion efficiency exceeding 10%, (ii) long‐term durability, and (iii) facile scalability. Indeed, the commonly used metal oxide semiconductors, such as TiO_2_, Fe_2_O_3_, BiVO_4_ and WO_3_, offer durability but demonstrate low efficiency; III‐V semiconductors, such as silicon (Si) and perovskites (PSK), could provide higher efficiency but are less durable (and often toxic) [3, 6, 7]. Due to the fast charge carrier recombination, poor light‐harvesting and electrode degradation observed for pure semiconductor photoanodes, composite hybrid materials (often hetero‐nanostructured) have been suggested where a semiconductor is integrated with noble metal plasmonic nanostructures [8, 9, 10, 11, 12, 13].

Historically, plasmon‐enhanced photoelectrochemical water splitting was first experimentally observed in 1996 by Yoko et al. [14]. Plasmonic catalysis involves excitation of localised surface plasmons (LSPs), which are the resonant collective oscillations of conduction electrons induced by photons [15, 16]. At LSP resonances (LSPRs), plasmonic nanostructures concentrate electromagnetic fields near their surfaces leading to a significant field enhancement. LSPRs occur at wavelengths dictated by geometry, metal type and dielectric environment of nanostructures and could induce a significant enhancement of the photocatalytic activity. Plasmon enhanced photoelectrochemical water splitting could potentially yield large efficiency. Moreover, it was theoretically predicted that the maximum enhancement of water splitting efficiency can be only achieved with the aid of plasmons [17]. Thus, plasmonic catalysis could be a promising avenue for enhancing the efficiency and selectivity of solar energy conversion [18]. Furthermore, plasmonic catalysts may have the capability to selectively activate chemical bonds of adsorbed molecules, thereby opening avenues for targeting specific chemical pathways within a chemical reaction [8]. Note that despite ever increasing interest in developing pure plasmonic photocatalyst, its efficiency is still quite low (∼0.1%) [13].

Plasmonic photocatalysis promise an improvement in efficiency due to the next four established mechanisms: (i) light absorption and scattering by plasmonic nanostructures, (ii) plasmon‐induced near‐field energy transfer (PIRET), (iii) hot‐carrier injection and (iv) local enhancement of the electromagnetic field (LEF) [15, 19, 20]. In hybrid plasmonic PEC water splitting, both semiconductors and metals in the plasmonic photoelectrode may interact with solar light. Plasmonic gold nanostructures (NSs) possess high chemical stability, efficiently absorb visible light (unlike many inorganic oxides) and enhance light–matter interactions with localised plasmons. Furthermore, in plasmonic photoelectrodes, gold NSs may improve the charge formation and energy transfer processes, which enhance the charge separation and improve light harvesting. The main disadvantages of noble‐like plasmonic metals, such as Ag and Au, are low intrinsic activities for molecule absorption due to their fully filled *d‐*bands and high costs [9].

On the other hand, non‐noble metals could be promising materials for NSs because they display strong LSPRs and are cost‐effective compared to noble metals [21]. Recent data reveal that the integration of non‐noble Al nanoparticles in the photoanode significantly improves PEC water splitting efficiency due to their unique optoelectronic properties, which include LSPR frequency tunability from UV to NIR region and high electron density. Notably, aluminium is the most earth‐abundant element behind oxygen and silicone. Moreover, Al can produce strong local electromagnetic fields due to low dc screening of Al (ε_∞_ ≈ 1) as compared to noble metals (silver ε_∞_ ≈ 4 and gold ε_∞_ ≈ 9) [22, 23]. For example, Li et al. fabricated plasmonic Al@TiO_2_ hetero‐structured photoanodes for PEC water splitting [24]. They showed that the composite photoanodes show higher photocurrent responses than pure TiO_2_ electrodes due to the presence of LSPRs of Al NSs that generate more hot electrons and amplify the electromagnetic field at the interface, facilitating charge separation and transport, thereby promoting efficient water splitting. However, aluminium is known to be a highly reactive chemical element reacting with ambient oxygen to create a protective coating called aluminium oxide (Al_2_O_3_) [25].

Plasmonics can affect several areas of photocatalytic water splitting through excitation of LSPRs. LSPRs provide enhanced electromagnetic fields and improved separation of splitting ions. While Al NSs are mostly effective in UV plasmonics, materials based on Al are effective in the visible and even infrared plasmonics [26]. The presence of LSPRs significantly improves light‐absorbing capacity by usage of all parts of light spectrum capable of water splitting, expedites the bulk and surface charge separation efficiency of nanostructured photoanodes and activates chemical bonds through enhanced electric fields leading to significant improvement in PEC performance. It is worth stressing that traditional semiconductors used as photoanodes (such as TiO_2_) utilise only UV part of the spectrum for water splitting which significantly restricts their efficiency.

Recently, we demonstrated that inexpensive MgB_2_ nanostructures can achieve efficient plasmonic water splitting and could be a viable alternative to noble metal nanoplasmonics [27, 28]. Aluminium diboride (AlB_2_) is similar to MgB_2_: it is stable in various environments and is facile to use in the fabrication process of building 2D nanosheets [29]. Moreover, the interband transitions between π and *σ* bands in AlB_2_ NSs, composed of *sp* ^2^ hybrid orbitals and *p*_*z*_ orbitals, promote more solar energy to be redirected for the excitation of LSPRs in comparison to the preferable *d*‐band interband transitions in the noble metals. It is natural to expect that LSPRs of AlB_2_ nanostructures could be more powerful for water splitting as compared to other diborides (MgB_2_, CrB_2_ and NiB_2_) due to higher concentration of free electrons and weaker electromagnetic screening.

Here, we show that inexpensive AlB_2_‐based photoanodes provide efficient plasmonic water splitting and could become a viable alternative to noble metal nanoplasmonics. We focus on the plasmonic effect in the hybrid AlB_2_‐based nanostructures and discuss its application in the photocatalytic production of hydrogen by solar light. The main reason for integrating other materials with plasmonic AlB_2_ nanostructures is to enhance light absorption through optical impedance matching (where the refractive index of photoanodes match that of the electrolyte) and to enhance water splitting efficiency through engineering of photoanode absorption to match the spectrum of solar light.

Using a mechanical rolling mill procedure, we engineered efficient light‐harvesting photoanodes with responsivity over a broad range of wavelengths. We show that LSPR field enhancement, plasmon‐induced electron transfers and improved conductivity of the hybrid photoanodes all could play roles in enhancing the efficiency of photocatalytic water splitting. With fabricated hybrid photoanodes based on AlB_2_, we were able to achieve ABPE efficiency of water splitting ∼10% under simulated solar illumination with an applied bias of 0.3 V, which is close to the commercial viability benchmark. We also achieved ∼5% ABPE efficiency of seawater splitting by suggested hybrid photoanodes and demonstrated their bifunctionality.

## 2 Results and Discussion

### 2.1 Plasmonic AlB_2_‐Based Hybrid Photoanodes for Water Splitting

The crystal cell of AlB_2_ is composed of two atomic layers: graphene‐like honeycomb borophene and triangular aluminium (Figure 1). It is worth noting that, in contrast to the bulk phase, monolayer AlB_2_ is predicted to be a superconductor with a high critical temperature [30]. Strong modifications of the electronic structure of low‐dimensional AlB_2_ NSs in comparison to the bulk form have stimulated significant research interest in the AlB_2_ family of hybrid nanostructures. Fabrication of the heterostructures in which plasmonic NSs are combined with transition metal dichalcogenide (TMDCs) nanosheets can be also considered as a new strategy to enhance the photocatalytic performance. Recently, our group reported on plasmonic MgB_2_/TMDCs metamaterials that exhibit superior photocatalytic activity due to the impedance matching, which enhances light absorption over the entire solar spectrum by the metal catalyst, as well as plasmon–exciton coupling [28].

**FIGURE 1:**
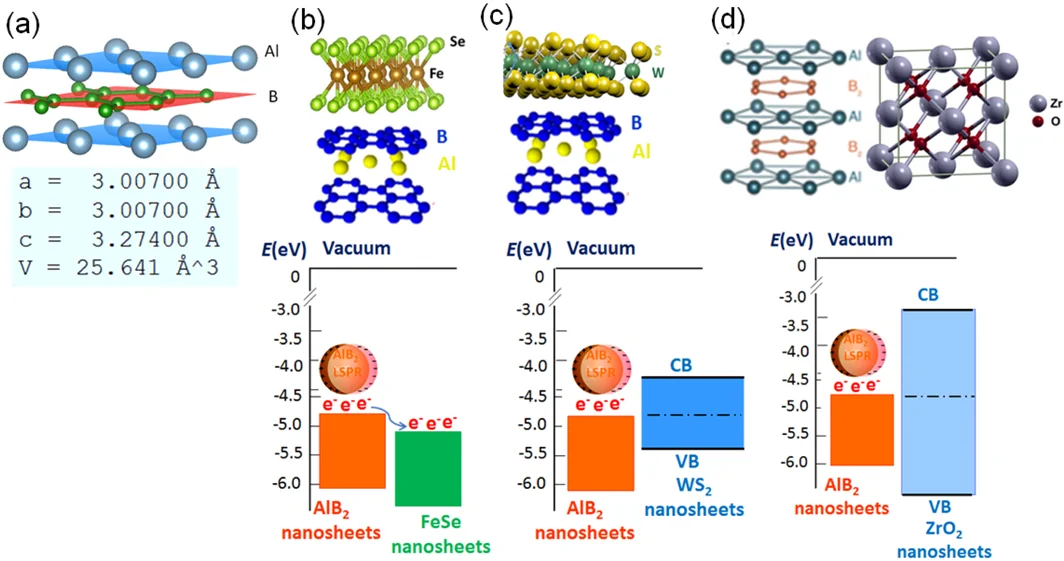
Plasmonic AlB_2_‐based photoanodes combined with various co‐catalyst nanomaterials: metallic FeSe, semiconductor WS_2_ and dielectric ZrO_2_. (a) The schematic models of the bare AlB_2_ consisting of graphene‐like honeycomb B layers with triangular packed Al layers. (b–d) The top panels show schematic structure of AlB_2_ with FeSe (WS_2_, ZrO_2_) nanostructures, respectively. The bottom panels display the schematic energy band structure of hybridised AlB_2_‐FeSe, AlB_2_‐WS_2_ and AlB_2_‐ZrO_2_ nanostructures based on the equilibria of the work functions for different nanomaterials, respectively.

This study demonstrates that coupling of LSPRs of closely spaced metallic NSs is significantly modified by the presence of other materials such as semimetal FeSe, semiconductor WS_2_ and dielectric ZrO_2_ (see Figure 1). The schematic models of the bare AlB_2_ photoanodes consisting of honeycomb B layers with close‐packed Al layers between them, and hybrid AlB_2_ with FeSe (WS_2_, ZrO_2_) photoanodes are shown in the top part of the Figure 1. Band models (bottom panel of Figure 1) are displayed for the AlB_2_ plasmonic NSs in the hybrid nanostructures by taking into account the close equilibrium in the work function for AlB_2_ (∼4.75 eV) and FeSe (∼5.1 eV) [31, 32], and also a small Schottky barrier formed at the AlB_2_‐WS_2_ interface (∼4.6 eV, work function of WS_2_) in contrast to a high Schottky barrier formed at the AlB_2_‐ZrO_2_ interface (∼3.3 eV, work function of ZrO_2_) [33, 34].

#### 2.1.1 Fabrication Procedure

To fabricate photoanodes with homogenously mixed AlB_2_ and co‐catalyst nanostructures, we used a rolling mill technique delivered by a Pepe Tools 90 mm Flat Rolling Mill. This technique enables mechanical exfoliation of the layered crystalline materials and sufficient adhesion of the fabricated layers to a copper foil used as the conductive substrates (we employed Cu foils with thickness of ∼100 μm and area of 20 mm × 20 mm). The procedure involved placing 30 mg of AlB_2_ and co‐catalyst powders, mixed at a 1:1 mass ratio, between two thin copper foils. AlB_2_, FeSe, WS_2_ and ZrO_2_ powders were purchased from Sigma Aldrich with purity of over 99%. It is worth noting that the resulting powder mixtures of AlB_2_ together with co‐catalyst nanomaterials were additionally mechanically grinded in a mortar and pestle. During the rolling mill procedure, the designed stacks Cu‐foil/AlB_2_‐2D‐NSs/Cu‐foil were passed multiple times (about 10) between rolls, while applied pressure continuously increased to obtain thin hybrid AlB_2_‐2D nanomaterial films. As a result, the samples were longitudinally expanded by a factor of ∼3. This method of fabrication introduces a new type of metamaterial photoanodes for effective photocatalysis and is described in detail in our recent works [27, 28].

We have tested the photocatalytic response of hybrid AlB_2_‐FeSe (WS_2_ and ZrO_2_) metamaterials with different AlB_2_ powder mass: 33, 50 and 67 wt. % mixed with corresponding FeSe (WS_2_ and ZrO_2_) flakes. The maximal photocatalytic efficiency was achieved for compounds of approximately equal masses of AlB_2_ and 2D atomic materials (see, Supporting Information S1: Figure S2). Accordingly, in this study, we have focussed on the physical and photochemical properties of 50% wt. AlB_2_‐2D material photoanodes. The rolling mill method could be suggested for the mass production of stable photoanodes on flexible large‐size substrates, providing effective photoanodes with multi‐wavelength LSPRs. Multi‐coloured optical images are shown below in Figure 2a–d. They confirm the excitation of LSPRs in AlB_2_ NSs. These images also show the presence of micrometre‐size flakes of the co‐catalyst (AlB_2_, FeSe, WS_2_ and ZrO_2_). Note that pure AlB_2_ nanostructures possess yellow‐greenish LSPR colours in contrast to more greenish LSPR colours for co‐catalyst nanostructures. The overall hybrid structures (for all metamaterials) appear to be black, which indicates a strong light absorption. The multitude of colours of the fabricated AlB_2_ and co‐catalyst 2D nanostructures is due to the change of reflectivity induced by excitation of LSPRs. The appearance of LSPRs (Figure 2) indicates that the fabricated samples should be described as optical “metamaterials” because the sizes of AlB_2_ and mixed AlB_2_‐FeSe (WS_2_ and ZrO_2_) building blocks are comparable to interrogating light wavelength. Such hybrid NSs strive to improve the light–matter interactions causing strong light absorption. As a result, they enhance the efficiency of photocatalytic water splitting as opposed to those achieved by assemblies of individual van der Waals flakes.

**FIGURE 2:**
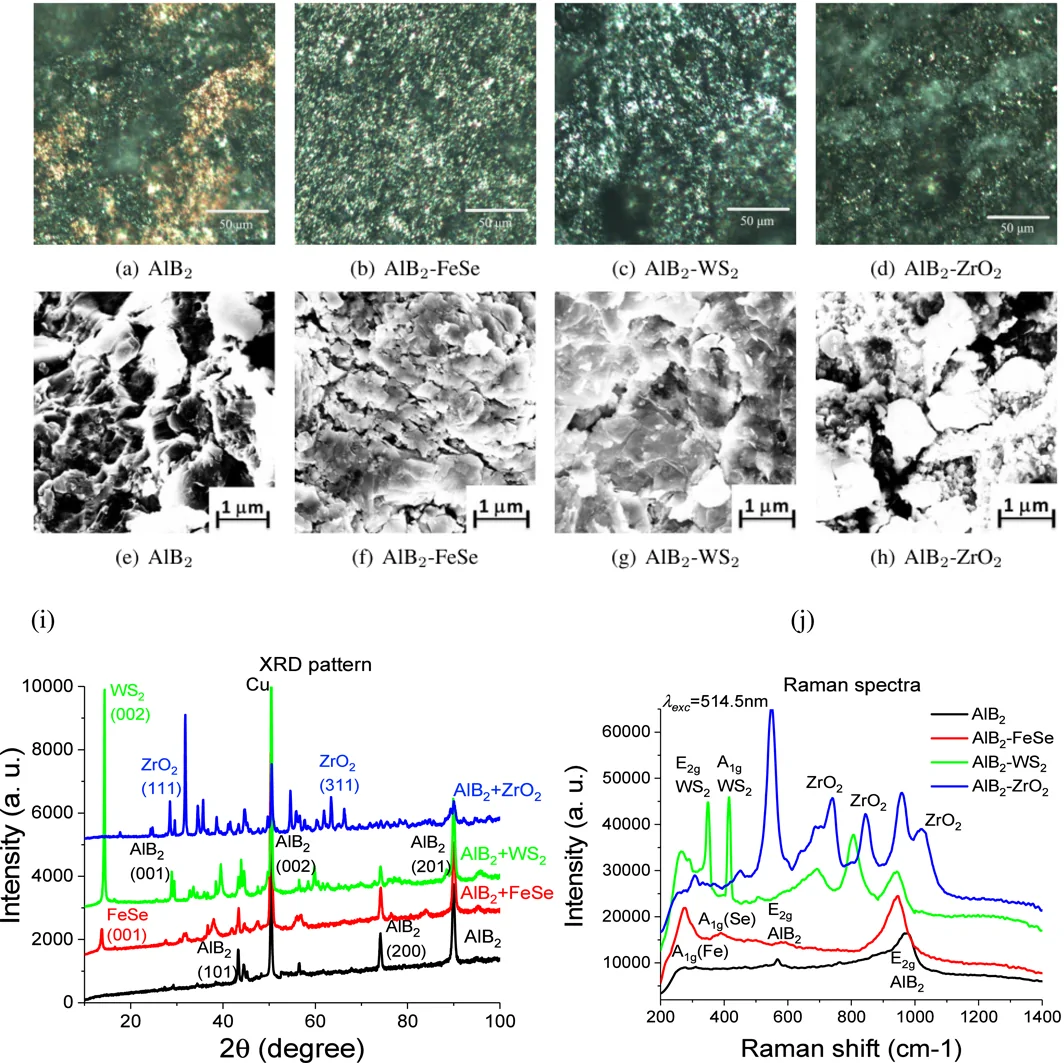
Optical microscopy and scanning electron microscopy (SEM) images of plasmonic AlB_2_ and hybrid AlB_2_ combined with FeSe (WS_2_, ZrO_2_) photoanodes produced by a mechanical rolling mill. (a–d) Optical images of pure plasmonic AlB_2_ and AlB_2_‐FeSe, AlB_2_‐WS_2_ and AlB_2_‐ZrO_2_ 2D‐like layered nanostructures. Optical microscopy images display the assembly of AlB_2_‐2D material photoanodes which exhibit different colours due to excitation of localised surface plasmon resonances. (e–h) SEM images of pure plasmonic AlB_2_ and AlB_2_‐FeSe, AlB_2_‐WS_2_ and AlB_2_‐ZrO_2_ 2D‐like layered nanostructures. (i) XRD analysis of hybrid nanostructured photoanodes. (j) Raman spectra of fabricated hybrid nanostructured photoanodes.

#### 2.1.2 Structural and Morphological Characteristics of Hybrid Metamaterials by SEM, XRD and Raman Analyses

High resolution scanning electron microscopy (SEM) images of mixed AlB_2_‐2D NSs hybrid metamaterials are shown in Figure 2e–h and Supporting Information S1: Figure S1. The SEM images demonstrate that fabricated samples consist of randomly mixed 3D‐like AlB_2_ blocks and flat 2D‐like FeSe, WS_2_ and ZrO_2_ NSs. It is worth noting that AlB_2_‐2D NSs hybrid metamaterials have a distribution of lateral dimensions from hubdreds of nanometres up to micrometres (0.1–5 μm) and a distribution of thicknesses from a nanometre to tens of nanometres (1–30 nm). In addition, narrow gaps of a few nanometres between different nanostructures were formed and produced strong interactions between the plasmonic AlB_2_ metal‐sheets and 2D NSs. The thicknesses of fabricated AlB_2_‐2D NSs hybrid photoanodes were approximately in range of 200–300 nm measured with the help of the mechanical profilometer Dektak 32 and extracted from data of spectral interference reflectivities.

X‐ray diffraction (XRD) was employed to identify the crystallinity and chemical structure of the AlB_2_‐2D materials hybrid photoanodes using CuKα radiation (*λ* = 0.15418 nm) in the range of angles 2*θ* from 10° to 100°. Figure 2i shows XRD spectra for the samples after water splitting experiments performed under *V*_bias_ = 0.3 V in the 0.5 M LiOH electrolyte (the results before/after experiments were almost identical which confirms high stability of fabricated photoanodes). There are diffraction peaks at approximately 44.5° and 56.5° in the XRD pattern for all investigated samples, corresponding to (101) and (002) planes of AlB_2_, respectively [35]. The AlB_2_‐FeSe exhibits characteristic diffraction peaks containing (001) and (002) planes, located at 13.8° and 33.0°, respectively, consistent with FeSe nanoflakes [36, 37]. The characteristic peak at 2*θ* *≈* 14.37° of the AlB_2_‐WS_2_ photoanode is clearly identifiable in XRD pattern (this peak belongs to the (002) planes of WS_2_) [38]. The additional peaks were observed for AlB_2_‐ZrO_2_ metamaterial photoanodes in the XRD pattern, see Figure 2i. For ZrO_2_ hybrid photoanodes, the primary characteristic diffraction peaks that appeared approximately at 2*θ* ≈ 30°, 35°, 50.8°, 60.1° and 63° corresponded to the crystal planes of (111), (002), (022), (311) and (222) [39]. Note that small shifts in the angle positions of the main diffraction peaks in the XRD pattern of AlB_2_‐2D material hybrid photoanodes occurred due to the internal tensile (compressive) strain (the analysis of the XRD patterns is presented in the Supporting Information S1: Table S1. Some additional peaks for AlB_2_‐2D hybrid photoanodes on Cu substrate could correspond to the Cu crystalline phases [40].

The Raman spectroscopy of hybrid AlB_2_—2D NSs photoanodes was conducted to determine the degree of crystallinity and the presence of defects in the metamaterials (Figure 2j). The Raman measurements were performed using a WITec confocal scanning Raman microscope under a 514.5 nm laser light and 1800 lines/mm grating with a 100× objective lens. The laser incident power did not exceed ∼0.5 mW to avoid damage or oxidation of AlB_2_ nanosheets. The main phonon peaks appear at 563 and 965 cm^−1^ for AlB_2_ photoanode [41]. The strong characteristic electron–phonon peak for nanostructured AlB_2_ arises from the E_2g_ phonon mode due to the in‐plane B‐B bond stretching. The Raman spectrum of AlB_2_‐FeSe photoanodes exhibits two discrete phonon modes in addition to those in the AlB_2_ sample (which are representative of FeSe): A_1g_(Se) at 387 cm^−1^ and B_1g_(Fe) at 282 cm^−1^ associated with lateral vibrations of the FeSe lattice [36]. Shifts in both phonon modes relative to the spectral position of the highly ordered FeSe structure can be seen as being due to the existence of internal tensile (compressive) strain. The level of strain developed in the layered FeSe is quantitatively comparable to the cases of multilayer MoS_2_ strained with the mechanically transferred processing technique [42]. Note that the first‐order Raman scattering from surface optical phonons for metals can arise due to the reducing symmetry at the surface. The present study shows that Raman scattering from these surface phonon modes can indeed be modified by surface defects and the interactions between AlB_2_ and FeSe resulting in new surface resonances in hybrid metals. Several phonon modes are observed for hybrid AlB_2_‐WS_2_ NSs in the Raman spectra at low wavenumbers, see Figure 2j. The two characteristic Raman modes, A_1g_ and E_2g_, corresponding to out‐of‐plane and in‐plane vibrations of W and S atoms, are observed at 414 cm^−1^ and 351 cm^−1^, respectively. We can identify other observed optical phonon modes with B‐B vibrations [43]. It is worth noting that the spectral separation between two A_1g_ and E_2g_ modes is characteristic of TMDCs layer thickness and provides an indirect way to assess the layer numbers [44]. The main phonon peaks appear at 547 and 960 cm^−1^ for AlB_2_ and 744, 849 and 1027 cm^−1^ for ZrO_2_ in the case of hybrid AlB_2_‐ZrO_2_ photoanode, respectively. Three peaks in the Raman spectrum of nanostructured ZrO_2_ are characteristic of the tetragonal phase of ZrO_2_ [45].

Scanning tunnelling microscopy (STM) was used to provide information about topography of the samples. STM images of several different areas of AlB_2_‐FeSe metamaterials photoanode are presented in Figure 3. STM images were measured at a bias voltage of 0.5 V and the set‐point current of 0.1 nA (the measurements were performed at the constant current mode). STM can characterise both structural morphology as well as local density of states. STM images (Figure 3) reveal that big flakes were composed of smaller crystallites merging together into larger islands. It is worth noting that the surface features observed in the STM images of Figure 3 closely resemble the corresponding morphological characteristics observed in SEM images (Figure 2). Compared to SEM, STM provides more information about the shape and thickness of the nanostructures formed on the surface of photoanodes. We see that majority of AlB_2_‐FeSe nanostructures exhibit thickness between 10 and 50 nm (Figure 3), while the average separation distance between of AlB_2_ and FeSe nanosheets is approximately 1–2 nm (Figure 3). These narrow gaps between different nanostructures indicate a strong interaction between the plasmonic metals and FeSe nanostructures. STM provides evidence that the rolling‐mill fabrication method favours the formation of two‐dimensional‐like nanostructures. This leads to the formation of elongated disk‐like nanostructures with thickness of 15–50 nm and nonsymmetrical sizes larger than 50 nm.

**FIGURE 3:**
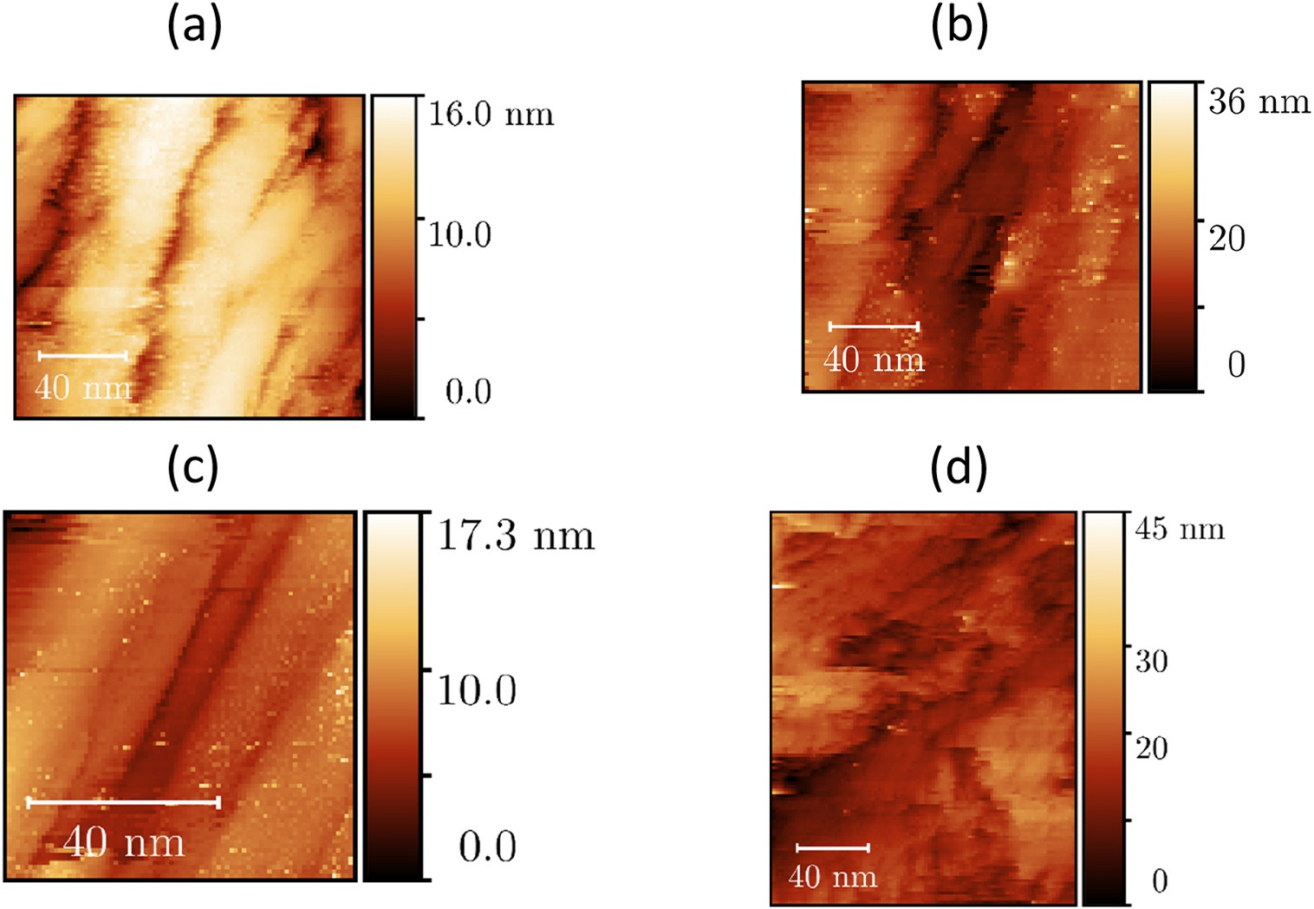
Scanning tunnelling microscopy images of different areas of AlB_2_‐FeSe metamaterials photoanode: (a–d). For the STM images, a voltage of 0.5 V was applied, and the set‐point current was set to 0.1 nA (the constant current mode).

#### 2.1.3 Photovoltaic Measurements

A PEC is composed of two electrodes immersed in an aqueous electrolyte and exposed to solar light. It is worth noting that photocatalytic water splitting could be compared to photosynthesis [46]. Natural photosynthesis is based on two photosystems: a photosystem I (PS‐I) and a photosystem II (PS‐II). Typically, in PS‐II, light active chlorophyll collects the solar energy to create photo‐excited electrons and holes. The resultant holes drive an oxidation reaction with the help of an oxygen‐evolving complex (manganese calcium oxide cluster), while the photo‐excited electrons are used by PS‐I for CO_2_ reduction [47]. In our PEC, oxidation reaction occurs at the anode surface leading to oxygen evolution, whereas reduction reaction occurs at the cathode surface providing hydrogen evolution. In this process, evolved H_2_ and O_2_ gases appear at two different electrodes. A detailed schematic of the used PEC can be found in [27, 28].

Photovoltaic performance of hybrid AlB_2_ and AlB_2_‐FeSe (WS_2_, ZrO_2_) photoanodes was measured using a solar simulator (Oriel Sol3A class AAA, Newport) and were calibrated using a silicon reference detector certificated by ThorLabs. The current‐voltage (I‐V) and current versus time (*I*(*t*)) characteristics AlB_2_‐2D materials were measured using the Keithley source meter (Keithley 2400) under simulated solar light AM 1.5 G conditions at an intensity of 100 mWcm^−2^ with and without an external potential bias (*V*_bias_) applied across the cell. The external potential bias is an applied voltage difference between a cathode and an anode, and it does not depend on the presence or the absence of a reference electrode.

Unfortunately, our installation did not allow us to collect hydrogen and oxygen gases. However, we did perform a calibration of current to gas conversion in our previous works (using the same setup), which showed 95% of Faraday efficiency—see also the discussion below. Hence, we can rely on the measured currents to evaluate the gas production with accuracy of 5% [48].

The performances of a typical plasmonic AlB_2_‐2D material photoanodes under illumination equivalent to 1 Sun and in the darkness are presented in Figure 4. The plasmonic AlB_2_ photoanodes generated photocurrents up to 6 mA/cm^2^ under *V*_bias_ = 0.3 V in 0.5 M LiOH electrolyte and ∼3 mA/cm^2^ in 0.5 M KOH electrolyte, respectively (Figure 4a,b). The measured photocatalytic activity was solely due to hybrid photoanode metamaterials and was not affected by a copper substrate which could be reactive in LiOH and KOH electrolytes. This was checked by measuring photocatalytic activity of pure Cu foils (substrates) in the analogous alkaline electrolytes. These measurements showed no change of photocurrent under illumination and the absence of electrocatalytic activity (no bubbles on anode and cathode).

**FIGURE 4:**
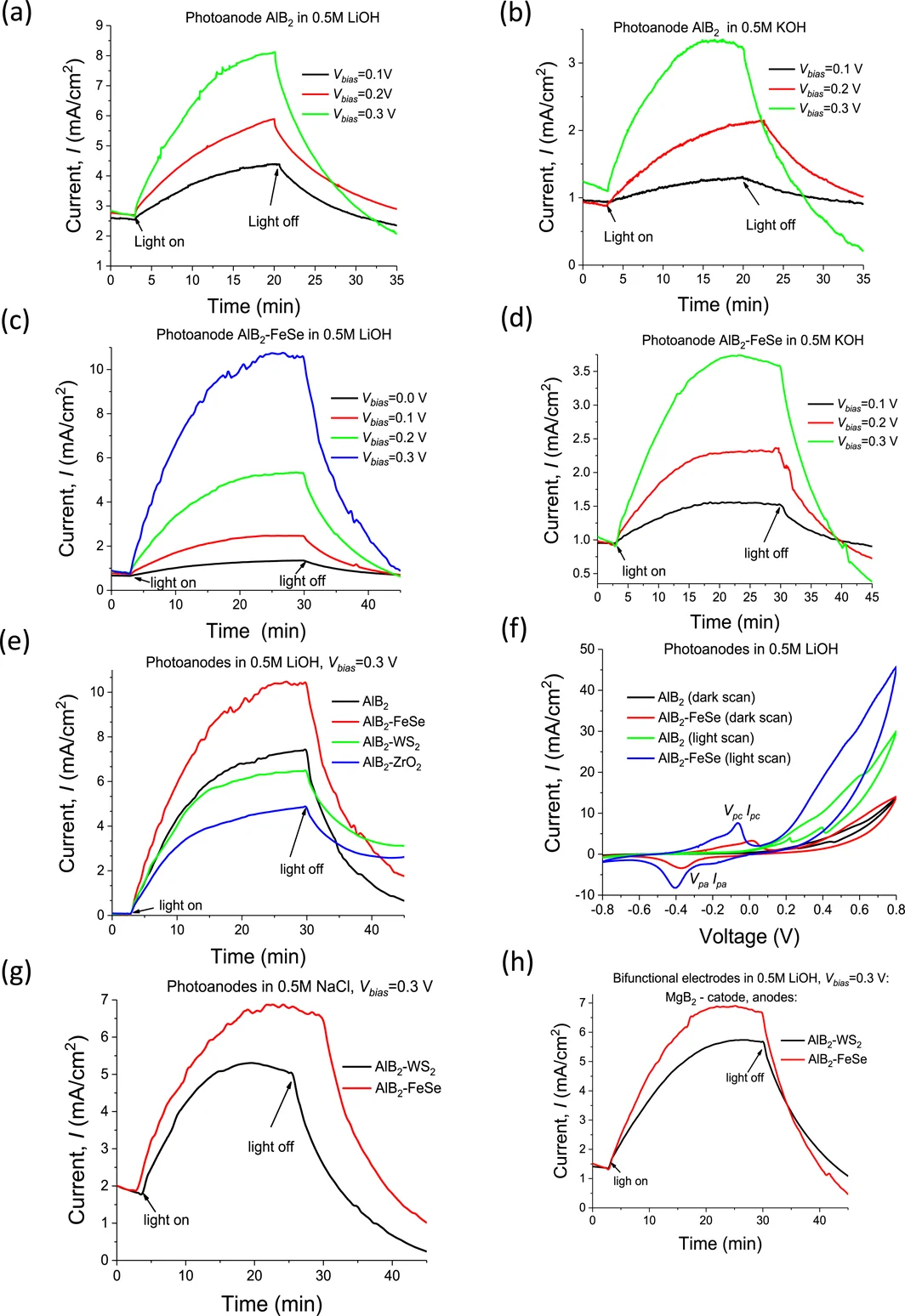
Solar water splitting. Photocurrent as a function of time and external voltage, *V*_bias_, for the integrated water splitting device under the illumination of a solar light simulator: (a, b) nanostructured AlB_2_ photoanode and the Pt cathode submerged in (a) 0.5 M LiOH electrolyte and (b) 0.5 M KOH electrolyte. (c, d) Nanostructured AlB_2_‐FeSe hybrid photoanode and the Pt cathode submerged in (c) 0.5 M LiOH electrolyte and (d) 0.5 M KOH electrolyte. (e) Comparison of the photocurrent as a function of time for different plasmonic AlB_2_ and hybrid AlB_2_‐FeSe, AlB_2_‐WS_2_ and AlB_2_‐ZrO_2_ photoanodes and the Pt cathode submerged in 0.5 M LiOH electrolyte. (f) Cyclic voltammetry scan of the investigated AlB_2_ and hybrid AlB_2_‐FeSe nanostructured photoanodes in the 0.5 M LiOH (compare dependences for dark and light scans). (g) Photovoltaic performance of the most stable and effective hybrid AlB_2_‐WS_2_ and AlB_2_‐FeSe photoanodes during the seawater splitting. (h) Demonstration of bifunctionality of the binary diborides metamaterials: AlB_2_‐WS_2_ and AlB_2_‐FeSe hybrid nanostructures working as a photoanode with nanostructured MgB_2_ working as a cathode.

In the next stage, we focused on the performance of AlB_2_‐FeSe, AlB_2_‐WS_2_ and AlB_2_‐ZrO_2_ hybrid photoanode, see photocurrent measurements and cyclic voltammograms presented in Figure 4. This allowed us to compare water splitting performance of metamaterials produced by combination of plasmonic AlB_2_ NSs with layered metal (FeSe), semiconductor (WS_2_) and insulator (ZrO_2_) NSs. Our champion device featured AlB_2_‐FeSe hybrid photoanodes and showed short‐circuit current density of 10.5 mA/cm^2^ for *V*_bias_ = 0.3 V in 0.5 M LiOH electrolyte (Figure 4c), and an applied bias photon‐to‐current conversion efficiency of ∼10%. In total, we tested more than 10 of AlB_2_‐FeSe photoanodes, with average ABPE being 9%–10%. PEC performance of AlB_2_‐FeSe photoanodes was also measured in a 0.5 M KOH electrolyte (Figure 4d). For KOH electrolyte, the difference between photocurrent in “on” and “off” states reached values of ∼2.9 mA/cm^2^. We also compared the performance of AlB_2_ and AlB_2_‐FeSe (WS_2_, ZrO_2_) metamaterials photoanodes in 0.5 M LiOH electrolyte (Figure 4e). We found that photocatalytic efficiency of AlB_2_‐FeSe had the highest efficiency value as compared to that of other nanostructured photoanodes. At the same time, AlB_2_ and AlB_2_‐WS_2_ showed approximately the same values of efficiency while AlB_2_‐ZrO_2_ photoanodes demonstrated the smallest water splitting efficiency.

It is worth noting that LiOH electrolyte provided almost doubled photocurrent as compared to seawater for the same AlB_2_‐FeSe photoanodes. This is connected to several factors: (i) different properties of the electric double layer for different electrolytes at the position where water splitting happens, (ii) better charge separation in alkaline solutions and (iii) different ion properties in different electrolytes.

The water splitting reaction by AlB_2_‐2D materials hybrid photoanodes was accompanied by bubbles formation on the surface of a photoanode (which generated O_2_) and a Pt cathode (which generated H_2_). The creation of the bubbles led to PEC photocurrent fluctuations and a slight drop of photocurrent at a longer timescale. The bubbles were seen by naked eyes. Optical images taken during the formation and growth of bubbles on the water splitting photoanode and cathode can be used to evaluate gas‐evolving reaction rate (for this procedure we periodically performed images of bubbles in PEC cell and counted their number). The conversion efficiency has been calculated using bubble microscopy approach [49]. We found that the overall volume of bubbles on the Pt photocathode was approximately two times larger than the overall volume of bubbles on the AlB_2_‐2D materials hybrid photoanodes, which should be expected from the water splitting reaction. Moreover, the overall volume of bubbles on both electrodes strongly increased with an increase of the bias voltage (and hence the photocurrent). Our previous measurements confirmed 95% Faraday efficiency of our installation [28, 48]. This implied that every two electrons generated ∼0.95 H_2_ molecules, and hence the ABPE represents overall efficiency of light conversion to hydrogen with 5%. It is worth noting that gas bubbles introduce additional optical losses by scattering incident light (reducing the photon flux reaching the photoanode) as well as reducing the access of water to photoelectrodes, thus decreasing photocurrent generation.

We used cyclic voltammetry (CV) to investigate the current‐voltage characteristics across the PEC cell for each type of photoanodes. Typical CV dependences for AlB_2_ and AlB_2_‐FeSe photoanodes are shown in Figure 4f and Supporting Information S1: Figure S3d (for AlB_2_‐WS_2_ and AlB_2_‐ZrO_2_). The photoanodes were tested in darkness and under solar light (AM 1.5 G, 100 mWcm^−2^) with a cycled applied voltage between −0.8 and 0.8 V (and in reverse) at a linear scan rate of 1.6 V/min. Under dark conditions, the measured current remained close to zero when total applied bias was below 0.5 V, which indicates negligible background electrochemical activity in this region. When switched to solar light illumination, unusual photocurrents were observed on the AlB_2_ and AlB_2_‐FeSe photoanodes. Moreover, the AlB_2_‐FeSe photoanodes exhibited substantially larger photocurrents than those of pure AlB_2_. This suggests that hybrid AlB_2_‐FeSe photoanodes could indeed play a favourable role in improving the PEC performance upon illumination. The shape of the I‐V curves is complex due to combined effects of diffusion and a rate of an electron transfer in photoelectrochemical process (Figure 4f and Supporting Information S1: Figure S3d). The important parameters of a cyclic voltammogram are the magnitudes of the anodic peak current (potential) *I*_*pa*_ *, V*_*pa*_ and cathodic peak current (potential) *I*_*pc*_, *V*_*pc*_ (Figure 4f). The number of electrons transferred in the photoelectrochemical reaction is proportional to the separation between the peak potentials, *ΔV* = |*V*_*pa*_ − *V*_*pc*_|. In our experiment, we have chosen the operating bias *V*_bias_ ≈ Δ*V*, which corresponds to the maximum stable photocurrent under illumination for efficiency comparison among investigated samples.

Relatively large dark currents displayed in Figure 4 before switching on illumination are associated with the existence of the initial contact potential established between plasmonic AlB_2_ or AlB_2_‐FeSe (WS_2_, ZrO_2_) metamaterial photoanodes and Pt cathode embedded in different electrolytes, which was measured at the level of *V*_init_ ∼0.7–0.9 V. This value of *V*_init_ originate from the difference in work function values between the photoanode and cathode. The time constant for reducing these currents to zero (due to formation of double layers and charge diffusion) was large in our installation (∼30 min) due to large separation between anode and cathode and a long time of charge diffusion between electrodes. At the same time, the changes induced by light illumination did not depend on the initial current, which we checked by waiting longer times before switching on the light.

#### 2.1.4 Seawater Splitting

There is a strong push to industrially produce H_2_ using seawater splitting because the oceans represent 96.5% of the total water reserves of the planet providing an almost unlimited resource [27, 28]. Unexpectedly, we observed an efficient room‐temperature seawater splitting by hybrid photoanodes based on plasmonic metal metamaterials made of aluminium diboride (AlB_2_) nanosheets combined with metallic FeSe nanoflakes (AlB_2_‐FeSe) or semiconductor TMDC co‐catalysts (AlB_2_‐WS_2_) under the light of a solar simulator. Our measurements demonstrate that these photoanodes remained stable in seawater environments and generate photocurrents up to 5–6 mA/cm^2^ under applied bias *V*_bias_ = 0.3 V, corresponding to a solar‐to‐photocurrent efficiency of ∼5% (Figure 4g). In these experiments, we modelled seawater as a solution of 0.5 M NaCl in deionised water (DI), where the measured pH of 0.5 M NaCl–DI water solution was ∼7−8, in agreement with ref. [50].

#### 2.1.5 Bifunctionality

Another important and unexpected property of fabricated plasmonic hybrid photoelectrodes based on metal‐boride nanostructures was their bifunctionality. In addition to providing efficient water splitting as photoanodes, they can also be used as a low‐cost alternative to expensive Pt cathode. Notably, the cost of platinum is more than two orders of magnitude greater than that of MgB_2_ [51]. We found that intermetallic (binary) diborides, such as MgB_2_ and AlB_2_, can indeed provide both the oxygen and hydrogen evolution reactions (OER and HER) in water. To show the bifunctionality of the studied materials, we have employed a MgB_2_ ring‐shaped cathode fabricated by the same rolling mill method as the AlB_2_‐based photoanodes instead of Pt. Figure 4h illustrates that the photocurrents of ∼5 mA/cm^2^ could be achieved under solar lights as a result of oxygen and hydrogen evolution reactions in the geometry solely based on binary diborides without the presence of Pt photocathodes.

#### 2.1.6 Ellipsometry Measurements

We used ellipsometry to measure optical properties of the produced photoanodes. It is well known that plasmonic metamaterials can absorb light over a wide range of wavelengths in case where NSs possess many LSPRs harvesting light energy efficiently [8, 9, 10, 48]. To show that we indeed have many LSPRs produced by NSs, we have performed variable angle spectroscopic ellipsometry measurements of AlB_2_ and AlB_2_‐FeSe (WS_2_, ZrO_2_) metamaterial photoanodes. The optical properties of hybrid AlB_2_‐2D NSs were measured in the reflection mode at three different incident angles (65°, 70° and 75°) using a variable angle spectroscopic ellipsometer (VASE) (J.A. Woollam Co.) in the 240–1700 nm spectral range. The experimental Psi (Ψ) and Delta (Δ) ellipsometric spectra were fitted using Complete EASE software [44, 52, 53]. The resonance peaks and dips in the optical spectra in Figure 5 are the results of specific morphology, that is, shape, size and configuration of AlB_2_ plasmonic nanostructures. As shown in Figure 5, the pure AlB_2_ photoanodes yields values of refractive index *n* in the range of 1.3–1.5 for wavelengths between 400 and 700 nm, closely matching the refractive index of the water, *n*_*wt*_ ≈ 1.33 providing impedance matching and reducing interfacing reflection losses. The presence of FeSe (WS_2_, ZrO_2_) NSs contributes significantly to the both the imaginary and real part of the effective refractive index *N* = *n* + *ik*, of hybrid metamaterials. The *n* and *k* increase monotonically after *λ* > 550 nm in positive direction for AlB_2_‐FeSe hybrid NSs and exhibit an excellent metallic response, represented by high *k* at long wavelengths. Similarly, the imaginary component of the effective refractive index (*k*) or material optical loss increases at long wavelengths due to free‐electron Drude absorption. In this case, we expect changes in the resonance of plasmon excitation and damping of the collective oscillation due to the increase in absorption. Note that the values of *n* measured for the AlB_2_‐FeSe samples suggest that they are very close to that of electrolytes—generally between 1.3 and 1.4 in the spectral range 400–900 nm, again indicating impedance matching and the absence of reflection.

**FIGURE 5:**
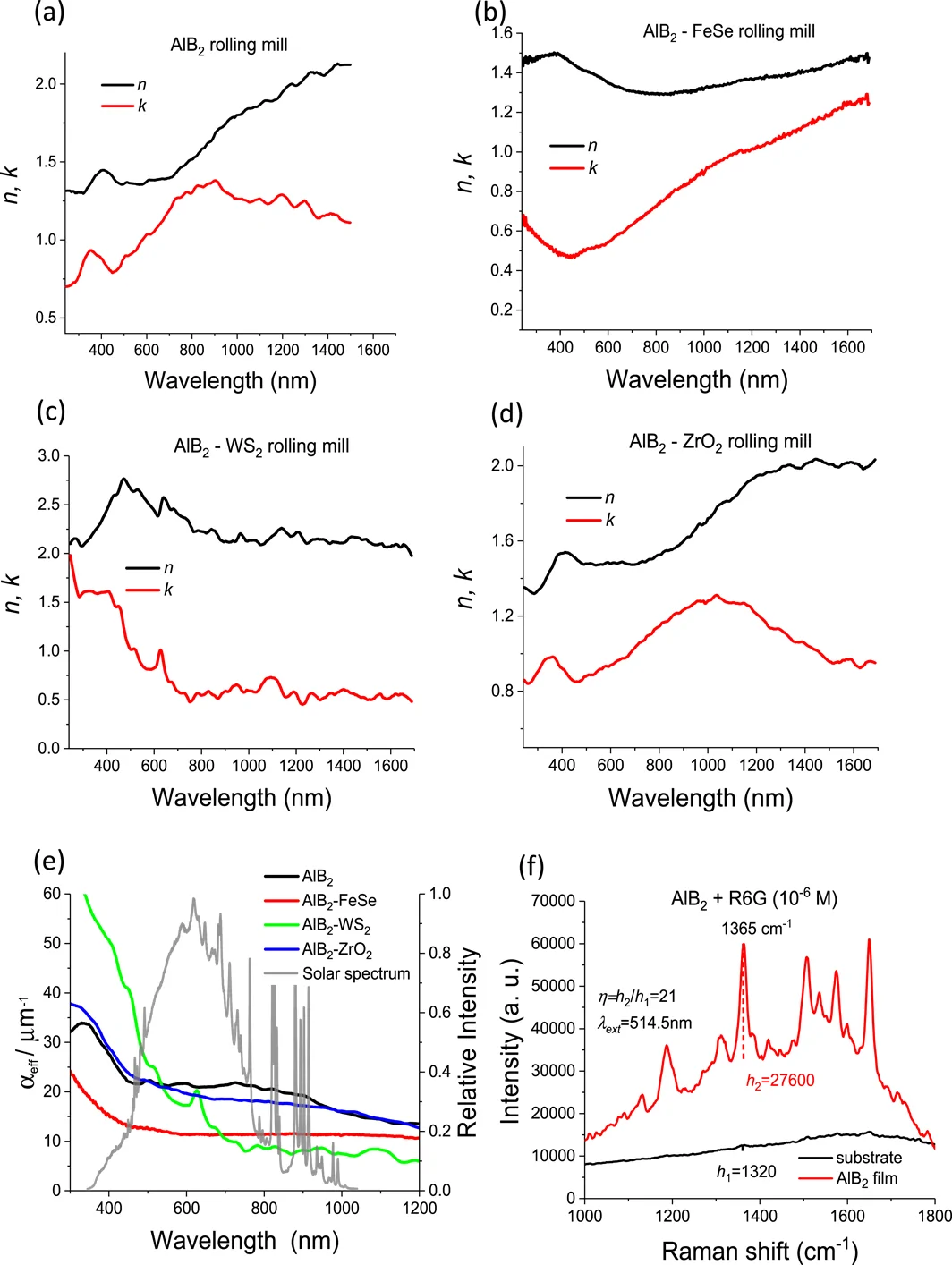
Optical properties of AlB_2_‐2D NSs films. (a‐d) The real and imaginary parts of the refractive index extracted for (a) pure AlB_2_ and (b) hybrid AlB_2_‐FeSe, (c) AlB_2_‐WS_2_ and (d) AlB_2_‐ZrO_2_ photoanodes. (e) Absorption spectra of a plasmonic AlB_2_ and hybrid AlB_2_‐FeSe, AlB_2_‐WS_2_ and AlB_2_‐ZrO_2_ photoanodes with normalised solar simulator AM 1.5 G spectral intensity. (f) Surface enhanced Raman signals from R6G dye molecules placed on top of AlB_2_ nanosheet assemblies due to LSPRs of metallic AlB_2_ nanostructures providing highly enhanced electromagnetic fields, measured at the excitation wavelength of *λ* = 514.5 nm.

The refractive index of the AlB_2_‐FeSe samples remains spectrally flat across large range of wavelengths and close to impedance matching which provides an explanation of the pronounced enhancement in photocurrent for these photoanodes. Combined with a reasonably small *k*, this leads to a small reflection from the water–photoanode interface and full absorption according to the Fresnel theory [54]. Excitonic effects play an important role in optical activity of TMDCs nanomaterials, which is evident from the appearance of optical peaks *n* and *k* in the visible region of AlB_2_‐WS_2_ hybrid photoanodes (Figure 5c) that coincide with the excitonic peaks in WS_2_. The A and B excitonic absorption peaks that arise from direct bandgap transition at K points were observed at 628 and 518 nm, respectively, for few‐layer WS_2_ [55]. There is also a substantial rise in absorption intensity that could be attributed to the excitation of LSPRs in AlB_2_. Moreover, a faint absorption peak exists at 455 nm, denoted as C in the absorption spectrum. ZrO_2_ is a broad bandgap (3.25–5.1 eV) semiconductor and it has been widely explored as a co‐catalyst due to the presence of a large number of oxygen vacancies on the surface, significant ion exchange ability and physicochemical stability. However, a high value of electric permittivity, a wide band gap and a low separation rate of photogenerated electrons and holes hinders wide‐scale applications of ZrO_2_ in photocatalysis [34]. To overcome this, various enhancement strategies like doping, heterojunction formation, incorporation of co‐catalysts, etc., have been potentially explored. We found that coupling ZrO_2_ with plasmonic AlB_2_ NSs could augment its photocatalytic performance. The data presented in Figure 5d demonstrate a negligible impact on both optical constants *n* and *k* in the case of an addition of ZrO_2_ co‐catalyst to the plasmonic AlB_2_ NSs (see Figure 5a,d). Note that the bandgap of a ZrO_2_ co‐catalyst is too high to facilitate significant electron transfer, as the potential barrier is large and thus requires tunnelling. Figure 5e shows the absorption spectra of a plasmonic AlB_2_ and AlB_2_‐FeSe (WS_2_, ZrO_2_) hybrid photoanodes together with normalised solar simulator AM 1.5 G spectral intensity. The solar photon flux was collected with a UV‐visible‐near‐infrared Ocean Optics USB4000 spectrometer using an optical fiber coupler. The absorption spectrum, *α*, was evaluated from the ellipsometric measurements of the optical constants *n*, *k*, using the equation *α* = 4π*k*/*λ* (where *λ* is the wavelength of the incident light). The plasmonic nature of absorption in AlB_2_‐based nanostructured films enables a greater overlap with the solar spectrum. Moreover, the spectral dependence of absorption spectrum, *α*, and its intensity for the bare plasmonic AlB_2_ photoanode clearly shows the best correlation with the spectral distribution of solar photon light.

#### 2.1.7 Surface‐Enhanced Raman Spectroscopy

Surface‐enhanced Raman scattering (SERS) was further employed to examine the localised electromagnetic field enhancement of hybrid photoanodes. Rhodamine (R6G) with a concentration of 10^−6^M in PMMA 950 (3% anisole solvent) was probed with the excitation wavelength of 514.5 nm for the Raman measurements. The entire hybrid NSs showed characteristic Raman peaks of R6G (Figure 5f, Supporting Information S1: Figure S5): the peaks at 1131 and 1189 cm^−1^ that belongs to aromatic C‐H bending. The other three peaks located at 1365, 1512 and 1615 cm^−1^ were attributed to achromatic C‐C stretching [56, 57]. According to the electromagnetic enhancement theory, the high SERS intensity of the AlB_2_ and AlB_2_‐FeSe (WS_2_ and ZrO_2_) metamaterials can be assigned to their strong LSPR effect. We have extracted the SERS enhancement factor (*η* = *h*_2_/*h*_1_)—the relative increase in the peak heights (intensities *h*_1_ and *h*_2_) from the photoanode samples as compared to the plain substrate. We found that the resonant Raman signal of R6G dye is enhanced by *η* ∼ 20 times in magnitude by near‐fields of LSPRs in the AlB_2_ nanostructures, confirming the occurrence of LSPRs. Furthermore, the SERS enhancement factor *η* decreases when the AlB_2_ NSs are embedded into a thin metallic FeSe, semiconductor WS_2_ and an insulating ZrO_2_ layer, Supporting Information S1: Figure S5. Moreover, the investigated photoanodes demonstrate strong colour diversities in the visible domain, which can be associated with LSPRs (Figure 2). It means that they possess excellent LSPR properties. The AlB_2_ NSs can also be used for improving water splitting performance in the visible and near‐IR light region. Note that smaller values of *η* ∼ 10 are exhibited by the AlB_2_‐ZrO_2_ photoanodes due to detrimental action of the oxides on AlB_2_ NS LSPRs.

Strong evidence of LSPRs excitation and plasmonic mechanism of photocatalytic seawater splitting in our samples can be deduced from enhancement of Raman scattering, see Figure 5f and Supporting Information S1: Figure S5. Optical absorption of the AlB_2_ NSs also demonstrates several peaks in the region of the 500–750 nm (Figures 5e and 6) that can be associated with excitation of LSPRs, while composite AlB_2_‐FeSe metamaterials showed high optical absorption even in near‐infrared region.

**FIGURE 6:**
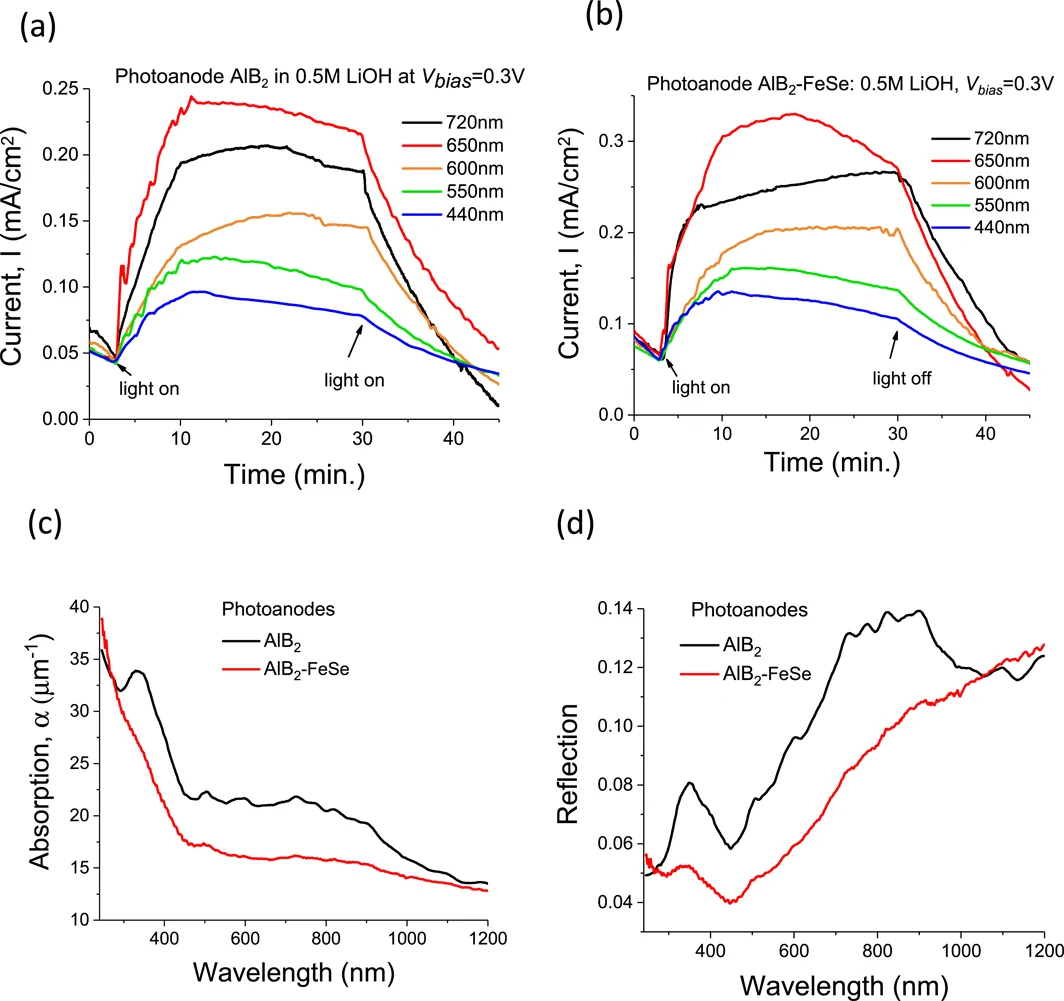
Photocurrent as a function of time for the water splitting device under the monochromatic illumination of a solar light simulator: nanostructured AlB_2_ (a) and AlB_2_‐FeSe (b) photoanode and the Pt cathode submerged in 0.5 M LiOH electrolyte. Absorption (c) and reflection (d) (in water condition) spectra of plasmonic AlB_2_ and AlB_2_‐FeSe nanostructures.

To study spectral dependence of water splitting, we have measured the photogenerated current under monochromatic sources of various wavelengths. Figure 6a,b show photocurrents generated by blue (*λ* ≈ 440 nm), green (*λ* ≈ 550 nm), orange (*λ* ≈ 600 nm) and red (*λ* ≈ 650, 720 nm) monochromatic light of approximately the same power density (∼10 mW/cm^2^) for AlB_2_ and AlB_2_‐FeSe nanostructures. Wavelength selection was done by applying a set of band pass filter (FWHM *Δλ* ≈ 20 nm, Thorlabs) using the solar simulator as the light source. The experiments were carried out in 0.5 M LiOH aqueous solution at pH∼13. These measurements show significant spectral increase of the effectiveness of photocurrent generation toward the near‐IR part of the solar spectrum. The monochromatic photon to current conversion efficiency (ABPE (*λ*)) of AlB_2_ (AlB_2_‐FeSe) under different wavelengths of light irradiation at 440, 550, 600, 650 and 720 nm are measured to be ∼1% (∼1.7%), 1.6% (2.2%), 2.1% (3.2%), 4.1% (5.5%) and 3.4% (4.1%), respectively, according to the data present in Figure 6a,b. We observe a maximum of power conversion efficiency of ∼5.5% under 10 mW/cm^2^ for *λ* ≈ 650 nm illumination. The evaluated ABPE (*λ*) for 650 nm is approximately 3–4 times larger than that for 440 nm (Figure 6). This increase is connected to the increase of number of near‐infrared photons for the same light intensity and LSPRs. The absorption band below 400 nm corresponds to intrinsic absorption (interband electron transitions) of AlB_2_‐based nanostructures. The excitation of LSPRs correspond to light absorption near 500 nm, 600 nm and 730 nm. Slightly less pronounced absorption peaks connected to LSPRs were also observed for AlB_2_‐FeSe nanostructures (Figure 6). It implies that AlB_2_‐based nanostructures sustain multiwavelength LSPRs and can efficiently absorb most of the sunlight spectrum. Reflection spectra confirm the impedance matching of the refractive index of the water, *n*_*wt*_ ≈ 1.33 and corresponding real part of ones for AlB_2_‐based nanostructures leading to reduction of reflection losses. Importantly, the FDTD results together with the effective‐film modelling demonstrated that AlB_2_‐FeSe photoanodes improve PEC water splitting by enhancing solar light absorption and producing a plasmon‐assisted near‐field response at the hybrid interface.

#### 2.1.8 Mechanisms of Plasmon‐Enhanced Catalytic Reaction in AlB_2_/2D Material Hybrid NSs

Optical measurements reveal that the integration of AlB_2_ NSs in the photoanode improves PEC water splitting efficiency due to their unique optical and electrical properties. Interestingly, the optical response of AlB_2_‐FeSe (WS_2_ and ZrO_2_) metamaterials can be tuned between UV to IR region as shown in Figures 5 and 6. AlB_2_ NSs with random sizes and distributions revealed hybridised LSPR effects to effectively absorb sunlight in wide wavelength ranges, while 2D‐like FeSe (WS_2_ and ZrO_2_) nanosheets provided an impedance matching that could efficiently reduce reflection. Plasmon resonances result in a strong oscillating electric field that surrounds AlB_2_ NSs and causes four primary mechanisms involved in photocatalysis such as (i) light absorbing and scattering by plasmonic NSs, (ii) plasmon‐induced near‐field energy transfer (PIRET), (iii) hot‐carrier injection and (iv) local enhancement of electromagnetic field (LEF) [8, 9, 10, 11]. The environment surrounding the AlB_2_ NSs also has a strong effect on the plasmonic resonance. The large dielectric constant of a medium around the plasmonic metal results in a more obvious red‐shift of plasmonic resonance (compare Figure 5a,d, for bare AlB_2_ and AlB_2_‐ZrO_2_).

Finally, we discuss plasmonic photothermal heating, which could contribute to the overall catalytic performance. We performed theoretical estimations of the temperature increase which predicted only a slight overall temperature increase on the surface of individual AlB_2_ nanoflakes at the level of Δ*T* = 5°C–7°C under solar irradiation. In contrast to electromagnetic fields, which could be well localised near NSs, temperature gradients are smoothed by high thermal capacity of water and reasonable thermal conductivity. We also experimentally checked that heating of water on hot spots is not responsible for the studied water splitting. First, we observed the formation of bubbles on both electrodes, where the bubbles on a smooth Pt wire cannot be explained by plasmonic LSPR effect. This also rules out chemical interaction of AlB_2_ or hybrid photoanodes with electrolytes as the reason for our measured data. Second, the overall number of bubbles on both electrodes strongly increased with an increase of the bias voltage, which would not be the case if the bubbles were produced by water heating induced by the solar simulator. Third, we performed experiments in a heated electrolyte with AlB_2_‐WS_2_ photoanode and Pt cathode, reaching temperatures up to 80°C. These experiments showed a decrease of efficiency of photocatalytic water splitting.

Photocatalytic response of AlB_2_ and AlB_2_‐FeSe (WS_2_ and ZrO_2_) metamaterials demonstrates slow rise upon illumination and slow decay for our PEC. This behaviour is connected to Helmholtz layers which appear near the surface of photoelectrodes. In an aqueous solution, water molecules (or ions in electrolyte) are attracted to electrodes due to their polar nature and produce the electric double layer. The double layer consists of the inner and outer Helmholtz layers which effectively screen any electric potential applied to the cell [58]. As a result, hydrogen ions (protons) produced during water splitting travel from a photoanode to a cathode by diffusion. Hence, the existence of the double layer within the PEC affects the time taken for the diffusion of a proton across the cell by negating the electric potential outside of it. In our geometry, the region outside of the double layer at each electrode makes up the vast majority of the distance the proton will travel due to diffusion. We can estimate rise (and decay) time, *t*, using diffusion equation $x^{2}=Dt$, where *x* ∼2 cm is the distance between electrodes and *D* is diffusion constant [58]. This gives a diffusion time of ∼10 min for our PEC system, which is in line with the measured values.

#### 2.1.9 Internal Electric Fields Between Al and B Layers

Large, permanent, internal electric fields are produced by the alternating layered structure of AlB_2_ with the Al layer being positively charged and the B layer being negatively charged. This happens due to the fact that chemical activity of Al is much larger than that of B and, therefore, each atom of Al layer transfers around 1.4 electrons (being a donor) to two atoms of B layer [59]. In the bulk, these fields just ensure charge separation of layers (positive charge for Al and negative charge for B). However, at the surface, they need to be screened. Since these electric fields strongly change at extremely small distances (separation between Al and B layers), they cannot be completely screened by Thomas–Fermi screening and, therefore, will affect electrolyte distribution (electrolyte double layer) near the sample surface [60]. Analogously, for MgB_2_, it was suggested that a Mg atom gives around a 0.2 e/Mg charge transfer to B‐plane, while for Al diborides, the charge transfer is expected to be 0.3 e/Al [29]. We can roughly evaluate the internal electric field between Al and B surface near the water edge as $E_{s}\approx \frac{\sigma }{2\varepsilon_{w}}$, where σ is the charge density transferred from one layer to another and $\varepsilon_{w}$ is the permittivity of first interfacial water layer. For AlB_2_, we have charge transfer of *q* ≈ 1.4 *e* per Al atom, and the lattice constant of *a* = 0.3 nm (Figure 1) [60], which yields very large electric fields between the layers as *E*_*s*_ ≈ 7 × 10^9^ V/m (∼7 V/nm). The internal electric fields are highly inhomogeneous near the surface of the metal where the catalytic reaction takes place. They provide an effective separation of ions produced by water splitting reaction as explained in refs. [28, 48].

The schematic model (Figure 7) demonstrates plasmon‐induced photocatalytic reactions under LSPR excitations and a LSPR‐mediated electron transfer process. In the first step, we should consider AlB_2_ not only as plasmonic NSs but also as well as having a 2D electronic structure which forms from the Al‐ and B‐terminated surface states. In contrast to graphene, however, the *σ* and π states derived from the boron orbitals in AlB_2_ can, and do, disperse in the out‐of‐plane direction. The level of out‐of‐plane dispersion is larger in AlB_2_ than in MgB_2_, according to the smaller *c/a* ratio (Figure 1a) in AlB_2_ [60]. This result in the energy difference between of the top of the *σ* holes bands and bottom of the π electrons bands reaching ∼1.1 eV [61]. In the case of photocatalytic reactions in the electrolytes with different pH we can use the following data: the hydrogen evolution reaction (HER) 2H_2_O + 2e^−^ → H_2_ + 2OH^−^, E_0HER_ = (0 − 0.059 × pH)V (vs. the standard hydrogen electrode (SHE)) and the oxygen evolution reaction (OER) 4h^+^ + 4OH^−^ → 2H_2_O + O_2_, E_0OER_ = (1.23 − 0.059 × pH)V (vs. SHE). Hybrid AlB_2_‐based NSs facilitate the water splitting due to several main mechanisms: (i) the excitation of its own LSPR, which is the resonances established between the electronic plasma and the incoming photons; (ii) interband electron transitions between the *σ*‐ and π‐bands in the neighbouring nanostructures and provoke electron–hole pair formation; (iii) LSPRs strongly enhance the electric field in the surroundings of the NSs, which accelerates electron transitions from the *σ*‐ and π‐bands due to the occurrence of the plasmon resonance energy transfer (PRET) [12, 27, 28]. The LSPRs of AlB_2_‐based photoanodes lie at around 500–800 nm, as evident from the dependence for the absorption coefficient *k* (Figures 5a,d and 6). It means that hot electrons generated from the LSPRs carried a maximum energy of ∼1.4–1.7 eV. Such energetic electrons from plasmonic AlB_2_ nanostructures can be injected into the π‐band of the terminated AlB_2_ surface nanosheets (Figure 7). The energetic diagram present was plotted including the data about work functions of the AlB_2_, FeSe and the redox potential of H^+^/H_2_ (*V*_*H+/H2*_ = −4.44 eV in DI (∼−5.1 V, in 0.5 M LiOH, pH ≈ 13) versus vacuum level, or ∼0.67 V relative to the standard hydrogen electrode (SHE)) and the redox potential of O_2_/H_2_O (*V*_*O2/H2O*_ = −4.9 eV in 0.5 M LiOH, pH ≈ 13) versus vacuum level, or ∼0.47 V relative to SHE in electrolyte with pH ≈ 13. The difference between the energies of the *σ*‐ and π‐bands exceeds the Gibbs free energy of water splitting in the case when the AlB_2_ and AlB_2_‐FeSe (WS_2_ and ZrO_2_) photoanodes and a Pt (MgB_2_) cathode are embedded into alkali electrolytes, meaning this energy is enough to split one water molecule into H_2_ and O_2_ [62]. We can summarise the main features corresponding to the effective water splitting based on hybrid AlB_2_‐FeSe (WS_2_ and ZrO_2_) NSs photoanodes: (i) absorption of light is enhanced by LSPRs of the hybrid photoanodes leading to increase of water splitting in the main AlB_2_ catalyst, (ii) these LSPRs induce intense electric fields and produce hot electrons in the corresponding WS_2_, ZrO_2_ semiconductor improving its co‐catalytic activity, (iii) AlB_2_ has negatively charged B layers and positively charged Al layers that provide strong local polarisation fields at exposed edge terminations, modulating the interfacial water orientation and facilitating charge transfer during water splitting. The work function of FeSe is larger than that of AlB_2_ NSs [32], which leads to an electrons transfer from AlB_2_ to FeSe surfaces. Thus, the charge transfer mechanism at the interface AlB_2_ and FeSe might be caused by bands bending (*σ, π* bands), which needs further confirmation in the future. Our direct comparison between photocatalytic efficiency of AlB_2_ and AlB_2_‐FeSe in this study stresses the important role of plasmon electrons transfer through of highly conductive metallic layers in the enhanced ABPE in the hybrid AlB_2_‐FeSe metamaterials.

**FIGURE 7:**
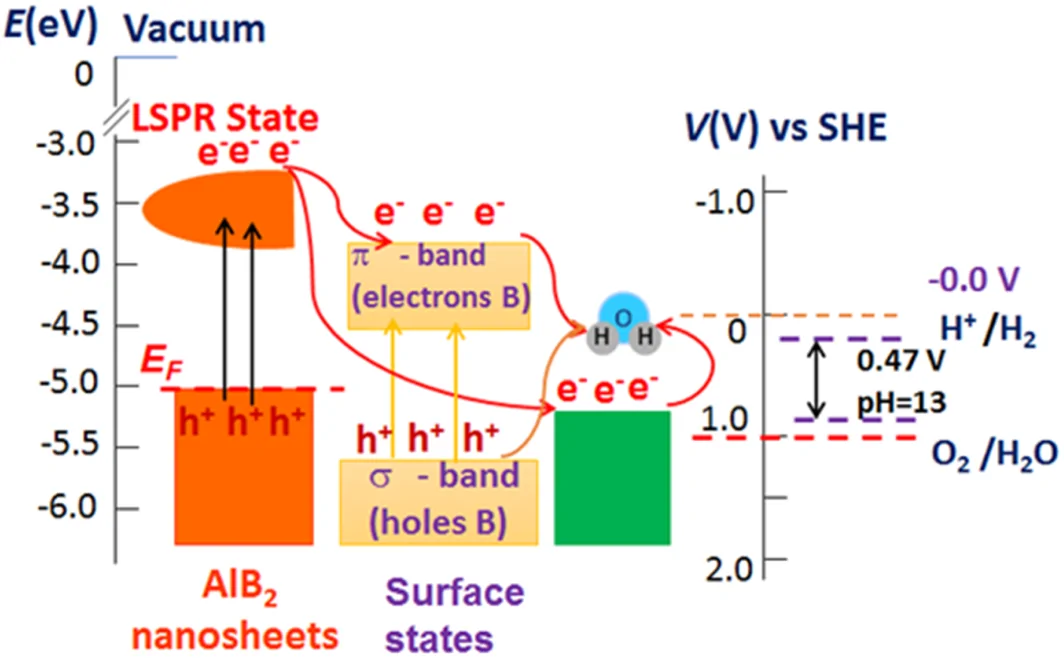
Schematic of charge distribution in diborides Al(Mg)B_2_ and the energy band diagram for plasmon‐enhanced water splitting based on AlB_2_‐FeSe hybrid photoanodes. Left: the Fermi level (E_F_) and hot electrons produced by LSPRs states for plasmonic AlB_2_ nanosheets. Middle: overview of the energy level positions of surface electrons in AlB_2_ nanostructures with respect to the work function. The top potential of surface electrons is more negative than the redox potential of H^+^/H_2_ (0 eV vs. SHE in the electrolyte with pH = 13) while the bottom potential of surface electrons (*σ*—like B) is more positive than the redox potential of O_2_/H_2_O (∼0.47 eV vs. SHE in the electrolyte with pH = 13). Right: the electron potential (in V) with respect to the redox potential of H+/H_2_ and redox potential of O_2_/H_2_O (in the case of electrolyte with pH = 13).

#### 2.1.10 Finite‐Difference Time‐Domain (FDTD) Simulations and Effective‐Film Optical Modelling

To support the optical origin of the photo‐response enhancement of AlB_2_‐FeSe, which is suggested by ellipsometry and SERS, we performed electromagnetic FDTD simulations using the experimentally measured optical constants. To this end, a transfer‐matrix effective‐film calculation was selected to estimate the optical response of the electrolyte/250 nm photoanode/Cu stack. FDTD simulations were performed for an idealised nanoscale AlB_2_ flake and an AlB_2_‐FeSe hybrid flake to evaluate the local field redistribution under representative wavelengths of visible and NIR regions. Disk‐like geometries with diameter of 100 nm and thickness of 30 nm for AlB_2_ and thickness of 20 nm for FeSe were used to simulate the flake material.

The effective‐film calculation showed that the mixed AlB_2_‐FeSe stack has very low reflection and high total absorptance over the visible‐NIR range which confirms the impedance matching achieved in the mixtures. For the electrolyte/250 nm film/Cu geometry, the mean total absorptance of AlB_2_‐FeSe reaches about 0.96 over 400–1200 nm, and the mean reflectance is only about 0.04 (Figure 8a,b). Note that the red/NIR region is particularly favourable. The calculated mean absorptance is about 0.98 and the mean reflectance is only about 0.03 over 700–900 nm range (Figure 8a,b). The values of mixed AlB_2_‐FeSe are higher than those of the pure AlB_2_ film and other mixtures in the same geometry, which is consistent with the highest photocurrent response of this composition.

**FIGURE 8:**
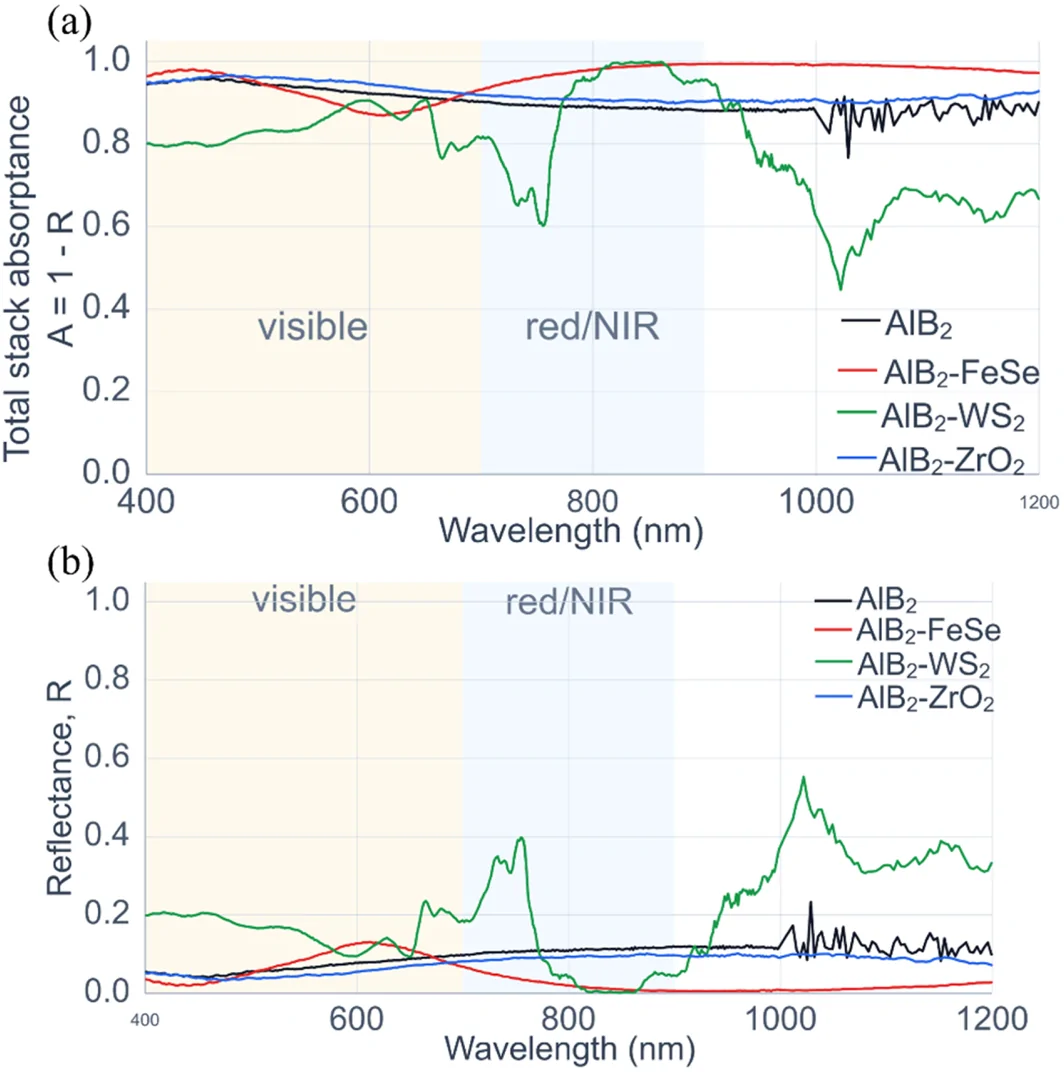
Effective‐film optical modelling of AlB_2_ and AlB_2_‐based hybrid photoanodes on Cu in water/electrolyte. The calculation assumes a water/electrolyte (*n* = 1.33)/250 nm effective film/Cu stack at normal incidence using ellipsometry‐derived n, k values for the photoanode films and Palik optical constants for Cu. (a) Total stack absorptance, *A* = 1—R. (b) Front reflectance, R.

The FDTD simulations allow one to see field distribution close to the nanostructures. The field factor F = |E_structure|^2^/|E_reference|^2^ is the field intensity enhancement induced by the AlB_2_ or AlB_2_‐FeSe nanostructures. Specifically, Figure 9a–d show the 500 nm simulation results (this wavelength is close to the Raman excitation wavelength used for SERS). We see that the field enhancement factor *F*_max_ = 1.2 for the pure AlB_2_ in the x‐z cross‐section increased to *F*_max_ = 1.3 for AlB_2_‐FeSe geometry, while in the x‐y gap‐plane section, the *F*_max_ increases from 1.03 to 1.26. At 900 nm, both pure AlB_2_ and AlB_2_‐FeSe nanostructures show strong edge‐field concentration. Still, the hybrid structure shows a higher *F*_max_ than pure AlB_2_ (Figure 9e–h).

**FIGURE 9:**
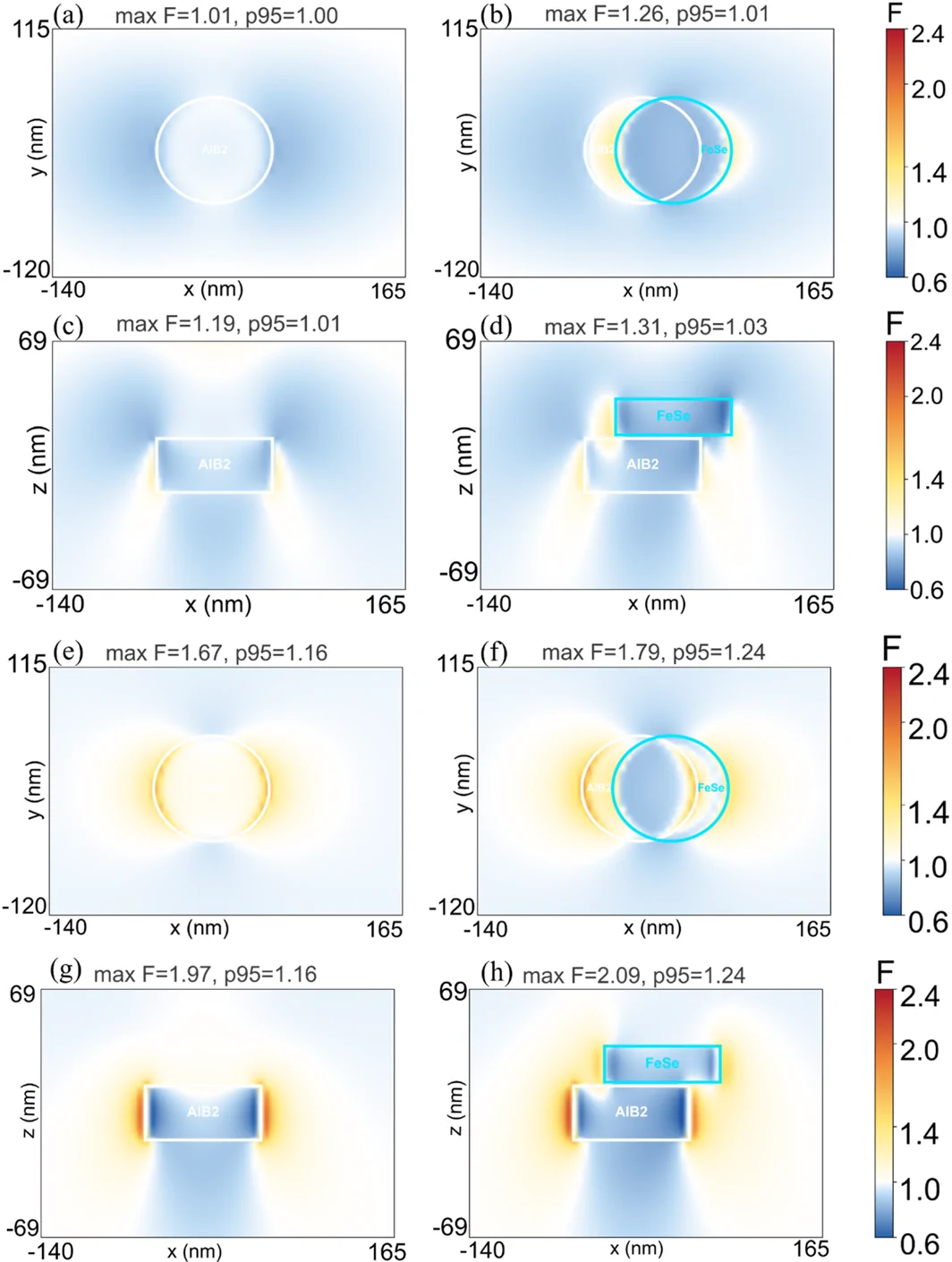
Reference‐normalised FDTD field maps for pure AlB_2_ and AlB_2_‐FeSe nanoflake geometries. F = |E_structure|^2^/|E_no‐structure|^2^, where the no‐structure reference was calculated using the same water/electrolyte background, source and monitors. p95 denotes the 95th percentile of the field factor over the monitor plane. White outlines represent AlB_2_ and blue outlines represent FeSe. (a–d) and (e–h) show the simulated field distribution at 500 and 900 nm, respectively. (a, b, e, f) x‐y field maps at the AlB_2_‐FeSe gap plane; for pure AlB_2_, the same z‐plane is used for direct comparison; (c, d, g, h) x‐z cross‐section maps. Pure AlB_2_ is shown in (a, c, e, g), and the AlB_2_‐FeSe hybrid geometry is shown in (b, d, f, h).

Although the calculated local field enhancements are moderate, the simulations clearly confirm the main idea of our work that the combination of metal catalyst with semiconductor (or dielectric) co‐catalyst can significantly reduce light reflection from photoanodes and improve the efficiency. At the same time, we observed that the introduction of FeSe can change the near‐field distribution and strengthen the field intensity at the AlB_2_‐FeSe interfaces and exposed flake edges. It is worth noting that the morphology of rolled films is more irregular. It contains rough edges, different flake shapes and nanoscale gaps, which could lead to other hotspots with enhanced fields. To conclude, the FDTD results together with the effective‐film modelling suggest that AlB_2_‐FeSe photoanodes improve PEC water splitting by enhancing solar light absorption and producing a plasmon‐assisted near‐field response at the hybrid interface.

## 3 Conclusions

We studied hybrid AlB_2_ photoanodes composed of metal catalyst (AlB_2_) and co‐catalysts made of metal, semiconductor and dielectric materials. The photoelectrodes were produced by low‐cost and high‐yield rolling mill technique and demonstrated excellent physical and chemical properties such as high optical absorption, large conductivity and stability, mechanical flexibility and high specific surface area. We showed that hybrid plasmonic AlB_2_‐FeSe photoanodes composed of metal catalysts and metal co‐catalysts exhibit the highest photocurrent density of ∼10 mA/cm^2^, which corresponds to an applied bias photon‐to‐current efficiency (ABPE) of 10%. This level of ABPE is close to the values required by industry to make photocatalytic water splitting viable technique for production of green hydrogen. We demonstrated that high efficiency of AlB_2_‐FeSe photoanodes is connected to (i) optical impedance matching since refractive index of the fabricated photoanodes matched the refractive index of electrolyte leading to very small reflection of incident light (in analogy with plasmonic blackbody) [63], (ii) reasonably large absorption coefficient of fabricated metamaterials which allowed us to achieve almost perfect absorption even for 200–300 nm films, (iii) matching of absorption coefficient spectrum of AlB_2_‐FeSe photoanodes with the spectrum of solar light. The plasmonic nature of photocatalytic water splitting in AlB_2_ and AlB_2_‐FeSe (WS_2_, ZrO_2_) hybrid photoanodes was confirmed by measuring the surface‐enhanced Raman scattering signals, which give clear evidence of the excitation of LSPRs in the AlB_2_ nanostructures. Seawater splitting using stable AlB_2_‐FeSe and AlB_2_‐WS_2_ photoanodes was realised and resulted in current densities of ∼4–5 mA/cm^2^ at solar radiation, which corresponds to ABPE of ∼5%. Finally, we showed that the studied hybrid photocatalysts can operate as bifunctional electrodes leading to current densities of 5–6 mA/cm^2^ at applied bias voltage of 0.3 V.

## Author Contributions

**Hongkai Zhou:** data curation, formal analysis, writing – original draft, investigation. **Andrew Campbell:** writing – original draft, formal analysis, data curation, investigation. **Wilfrid Harvey:** writing – original draft, data curation, formal analysis, investigation. **Vasyl G. Kravets:** conceptualization, methodology, investigation, validation, writing – original draft, writing – review and editing, project administration, supervision. **Alexander N. Grigorenko:** conceptualization, methodology, investigation, validation, writing – original draft, writing – review and editing, project administration, resources, supervision, funding acquisition.

## Conflicts of Interest

The authors declare no conflicts of interest.

## Supporting information


Supporting Information S1

## Data Availability

The data that support the findings of this study are available on request from the corresponding author. The data are not publicly available due to privacy or ethical restrictions.
