# Supplementary material for: Hybrid Plasmonic Photoanodes for Effective Photocatalytic Water Splitting
Source: Nanophotonics. 2026 Aug 13;15(16):e70237. doi: 10.1002/nap2.70237 (PMC13672165; doi:10.1002/nap2.70237)
Supplement: Supplementary file 1 [file NAP2-15-e70237-s001.pdf]

Supporting Information

for

Hybrid plasmonic photo-anodes for efficient photocatalytic water splitting

*Hongkai Zhou, Andrew Campbell, Wilfrid Harvey, Vasyl G. Kravets, Alexander N. Grigorenko**

Department of Physics and Astronomy, the University of Manchester, Manchester, M13 9PL, UK

E-mail: sasha@manchester.ac.uk

Sample fabrication. Photo-anodes for a photo-electro-chemical (PEC) cell were made on a copper foil with thickness of 100 µm covered by AlB_2 or hybrid AlB_2-FeSe (WS_2 and ZrO_2) thin nanostructures. To fabricate hybrid nanostructures we utilised the Pepetools 90 mm Flat Rolling Mill. At the beginning of the fabrication process, we covered the surface of a Cu foil of size 20x20 mm^2 by AlB_2, or mixed AlB_2 –FeSe (WS_2 and ZrO_2) diboride powders, weighing approximately 30 mg. All powders were purchased from Sigma Aldrich and had a purity of greater than 99%. AlB_2 and co-catalyst powders were mixed at a 1:1 mass ratio. The resulting mixing powders of AlB_2 and co-catalyst nanomaterials were additionally mechanically grinded in a mortar. During the rolling mill procedure the stacks of Cu-foil/AlB_2-2D-film/Cu-foil were passed multiple times (about 10) between rolls, while the applied pressure was continuously increased in order to obtain thin hybrid AlB_2-2D material films. This procedure leads to the lateral expansion of the samples by a factor of 3, giving a final size of 30mm x 40mm.

Characterisation of the photo-anodes

X-ray diffraction (XRD) was employed to identify the crystallinity and chemical structure of the AlB_2 - 2D NSs hybrid photoanodes using CuKα radiation (λ = 0.15418 nm) in the range of angles 2θ from 10° to 100°. Main Figure 2i shows XRD spectra for the samples after water splitting experiments performed under V_{bias}=0.3 V in the 0.5M LiOH electrolyte (the results

before/after photo-voltaic experiments were almost identical). Detailed analysis of the XRD patterns is present in the Table 1 below.

Scanning electron microscope images. An Ultra Plus Carl ZEISS scanning electron microscope (SEM) was used for high-resolution imaging of nanostructures. (The employed SEM has a technology for charge compensation that enables high resolution and provides stable and noise-free images from nanostructured samples as well as information about the samples' surface morphology.) A unique in-lens SEM detector gives resolution of the order of 1.0 nm at 15 kV (1.6 nm at 1 kV), dependent on the type of samples.

SEM images of mixed AlB_2 - 2D NSs hybrid metamaterials are shown in main Figure 2e-h and Figure S1a-d. The SEM image demonstrates that fabricated samples consist from randomly mixed 3D–like AlB_2 blocks and flat 2D–like FeSe, WS_2 and ZrO_2 NSs. It is worth noting that AlB_2 -2D NSs hybrid metamaterials have a distribution of lateral dimensions from tens of nanometers up to micrometers (0.1-5 μm) and a distribution of thicknesses from a nanometer to tens of nanometers (1-30 nm). In addition, narrow gaps of few nanometers between different nanostructures were formed providing strong interaction between the plasmonic AlB_2 metal-sheets and 2D NSs. The thickness of fabricated AlB_2 - 2D NSs hybrid photo-anodes was approximately in range of 200-300 nm measured with the help of the mechanical profilometer Dektakt 32 and extracted from data of spectral interference reflectivities.

Table 1. The data of X-ray diffraction (XRD) patterns of the crystallinity and chemical structure of the AlB_2 - 2D NSs hybrid photo-anodes using CuKα radiation (λ = 0.15418 nm) in the range of angles 2θ from 10° to 100°.

Samples	Diffraction peaks, 2θ (degree)	Crystalline planes	Literature
AlB_2	44.5°, 56.5°,61.7°, 67.9°, 72.5°, 79.2°, 88.5°	(101), (002), (110), (102), (200), (201), (112)	[S1]
AlB_2 - $FeSe_2$	$FeSe_2$: 13.8°; 33.0°, 54.09°	(001) FeSe, (002) FeSe, (0 3 1) $FeSe_2$	[S2, S3]
AlB_2 - WS_2	WS_2: 14.37°	(002) WS_2	[S4]
AlB_2 - ZrO_2	ZrO_2 : 30°, 35°, 50.8°, 60.1°, 63°	(1 1 1), (0 0 2), (0 2 2), (3 1 1), (2 2 2) ZrO_2	[S5]

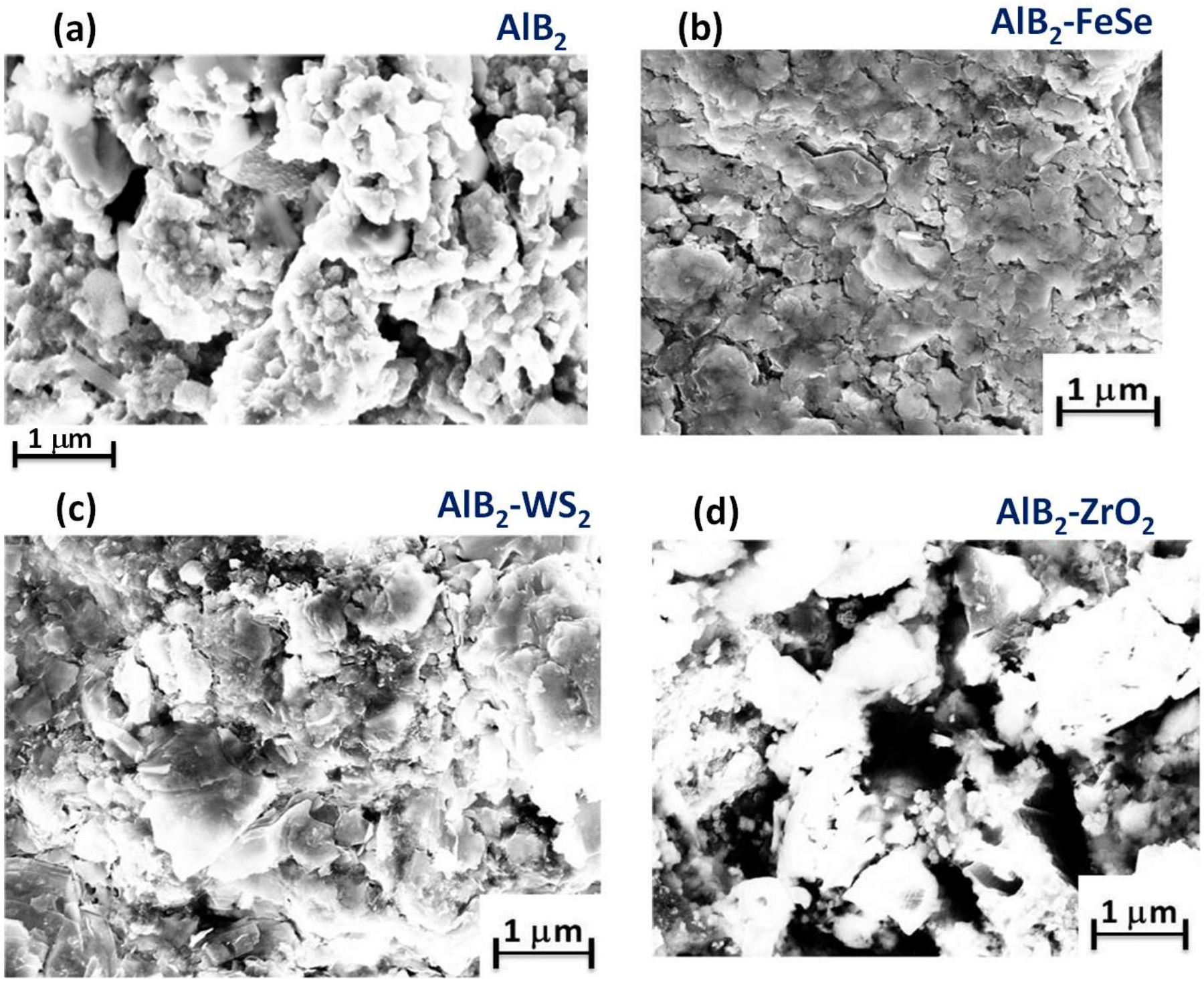

Figure S1. Scanning electron microscopy (SEM) images of plasmonic (a) AlB_2 (b) AlB_2-FeSe (c) AlB_2-WS_2, (d) AlB_2-ZrO_2 hybrid nanostructures.

Photocatalytic water splitting. The photocatalytic H_2 production was performed in a 50 ml cylindrical polymer reactor with height 5 cm and diameter 2 cm. AlB_2 or hybrid AlB_2-FeSe (WS_2 and ZrO_2) photo-anodes fabricated on Cu substrates with a size approximately 10 x 10 mm^2 were submerged in ~20 ml of various solutions: DI water, LiOH, KOH, NaCl electrolytes (the last one was used to imitate seawater). The cathode consisted of a spiral formed Pt wire (or a thin ring of MgB_2 for bi-functional measurements) with an area of approximately 0.15 cm^2. Such spiral design allows one to maximise the intensity of the incident light from the solar simulator to pass through and reach a hybrid photo-anode. An AlB_2 or hybrid AlB_2-FeSe (WS_2 and ZrO_2) photoanode and the Pt cathode were both immersed in an electrolyte solution. Subsequently, the reactor was illuminated by a solar light simulator (Oriel Sol3A, AM 1.5, 400 W Xe, ~100 mW cm^{-2}). To separate the two electrodes and don't block gathering bubbles we used the dielectric rubber ring of 3 mm thickness. The resulting current was collected using a Keithley 2400 sourcemeter and recorded using a LabVIEW programme. The produced photocurrent steeply increased after light was switched

until it reached its photochemical equilibrium (the plateau on the Figure 3) and then again steeply decreased in dark condition (when the light was turned off).

Figure S2 shows photocatalytic response of hybrid AlB_2-FeSe metamaterials with different AlB_2 mass ratios: 33 (1:2), 50 (1:1) and 67 (2:1) wt. % mixed with corresponding FeSe nanosized flakes. The maximal photocatalytic efficiency was achieved for compounds with approximately equal masses of AlB_2 and FeSe nanostructures. The measurement of photocurrent response of AlB_2-based nanostructures was accompanied by the bubble formation on the surface of the photo-anode (which generated O_2) and the Pt (which generated H_2). The bubbles were seen by naked eyes. The overall volume of bubbles on the Pt wires was approximately two times larger than the overall volume of bubbles on the AlB_2-based photo-anode which corroborates water-splitting reaction. Moreover, overall number of bubbles on both electrodes strongly increased when an increase of the bias voltage which confirms the water splitting and H_2 generation.

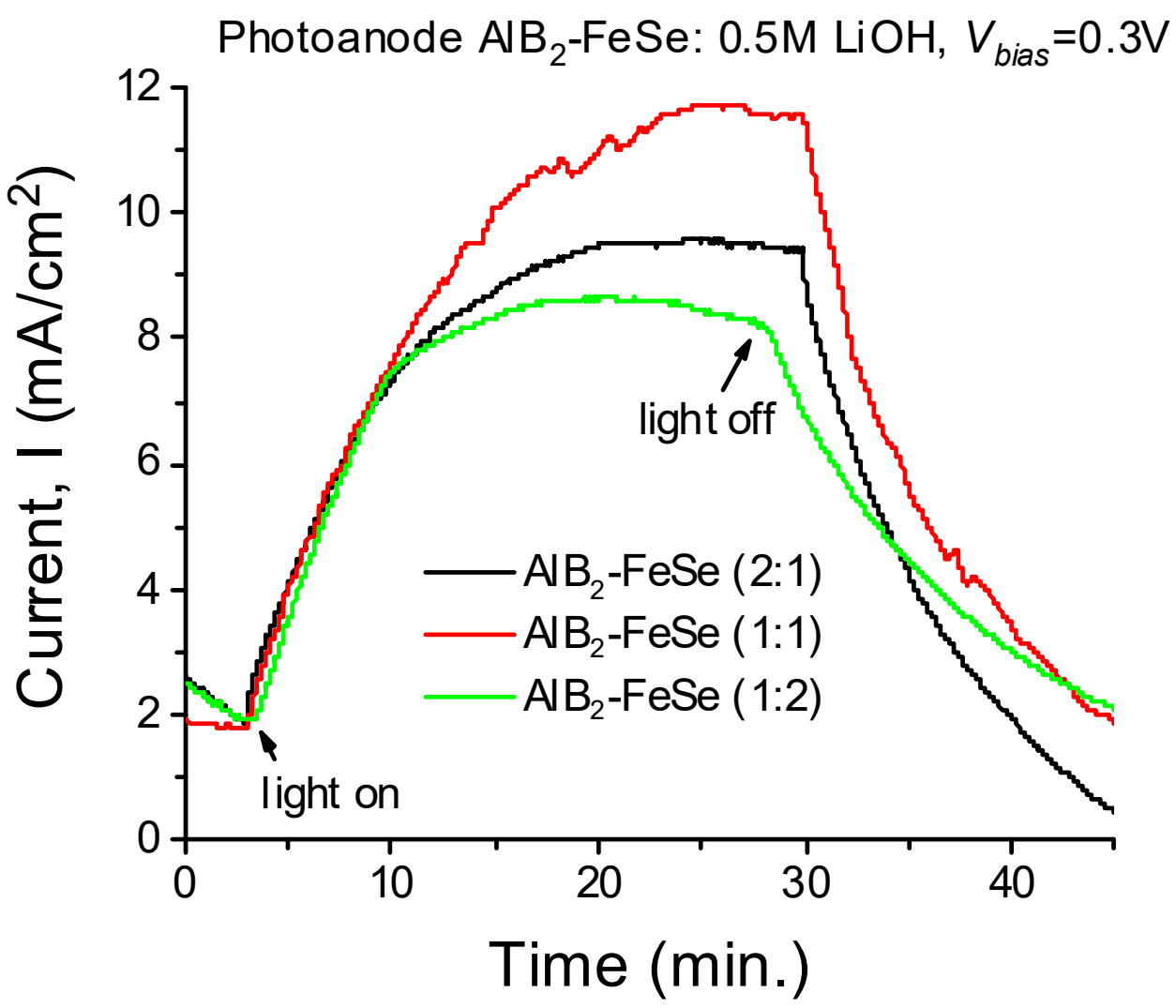

Figure S2. Photocurrent as a function of time for nanostructured AlB_2-FeSe photoanode with different concentrations of plasmonic metal AlB_2 and FeSe nanosheets with Pt cathode submerged in 0.5M LiOH electrolyte under external voltage, V_{bias}=0.3 V.

Stability and reproducibility of PV measurements. To assess reproducibility and stability of AlB_2 and AlB_2-FeSe photo-anodes, we examined the photocurrent generated by the different samples after immersion in 0.5M LiOH electrolyte. Plasmonic AlB_2 photoanodes (Figure S3a) showed variation in photocatalytic capability of approximately 15-20% between different samples. Different AlB_2-FeSe photoanodes exhibit similar 15-20% variation of

measured photocurrents as pure plasmonic AlB_2 ones (Figure S3a,b). Such distribution in the photocatalytic activity for different samples was caused by variations in structure of AlB_2 and AlB_2-FeSe photoanodes caused by different internal tensile (compressive) strains in the nanostructures. Shifts in the both phonon modes respect to the spectral position of highly ordered AlB_2 and FeSe-AlB_2 nanostructures as results of existence of internal tensile (compressive) strains were induced during rolling mill sample fabrications and confirmed by recording Raman spectra (main Figure 2j). We also examined the photocurrent generated by the AlB_2-FeSe photoanodes after immersion in 0.5M LiOH electrolyte for 0, 4 and 8 hours (see Figure S3c). In this experiment the photocurrent was allowed to saturate, after which the solar lamp was switched off and the system was monitored until the current returned to its original value. The FeSe-AlB_2 photoanodes demonstrated a gradual reduction in photocatalytic capability of approximately 7% between the first and second measurements, while, between the second and third, the decrease was more pronounced at around 10% (resulting in an overall 15% decrease). We suspect a significant contribution to this loss in the output is due to insufficient adhesion of some of the nanostructures to the copper substrates, as we directly observed macroscopic black flakes remaining at the bottom of the PEC cells. Plasmonic AlB_2 photoanode stability measurements yielded similar results to the AlB_2-FeSe photoanodes. This trend was observed multiple times for different hybrid AlB_2-based 2D nanostructures. It was found that the most effective AlB_2-FeSe photoanode remained active after 48 hours of 0.5M LiOH water splitting and stayed stable during the long-term photocatalytic activity. Note that after the first measurement, the dark current contributions reduced significantly, further suggesting saturation of surface reactions, resulting in a quasi-stable state.

Linear sweep voltammograms (LSV) of AlB_2 and AlB_2-FeSe (WS_2, ZrO_2) photo-anodes in 0.5M LiOH electrolyte were tested in dark and light illumination (Figures 4 and S3d, AM 1.5 G, 100 mWcm^{-2}) by continually cycling bias voltage between −0.8 − 0.8 V, and in reverse, with a linear scan rate of 1.6 V/min. When switched to light illumination, the significant increase of photocurrent was displayed on the AlB_2-2D hybrid photoanodes. Note that AlB_2-FeSe photoanode showed substantially larger photocurrent than that of other NSc metamaterials, which is almost 2 times the photocurrent of the AlB_2-ZrO_2 at 0.8 V and for large V_{bias}=0.7-0.8 V the values of current for AlB_2-WS_2 exceed that for the bare AlB_2 (Figures 4 and S3d). Furthermore, the slopes of the cyclic voltammograms (in Figure S3d) verify the onset of the HER even at zero bias. Additionally, we verified the role of AlB_2 as the primary catalyst and FeSe, WS_2 and ZrO_2 as co-catalysts of hybrid photoanodes because

the cocatalysts alone showed very little photo-response (< 2 mA) as compared to the hybrid photoanodes. In the case of a 2-electrode system (anode and cathode), the voltage in the *x*-axes corresponds to the bias applied to the whole device. Obtained data in Figures 4 and S3d suggest choosing the bias voltages which correspond to the point where the peak current is observed to providing the most effective value for the maximising efficiency of the photocatalytic process.

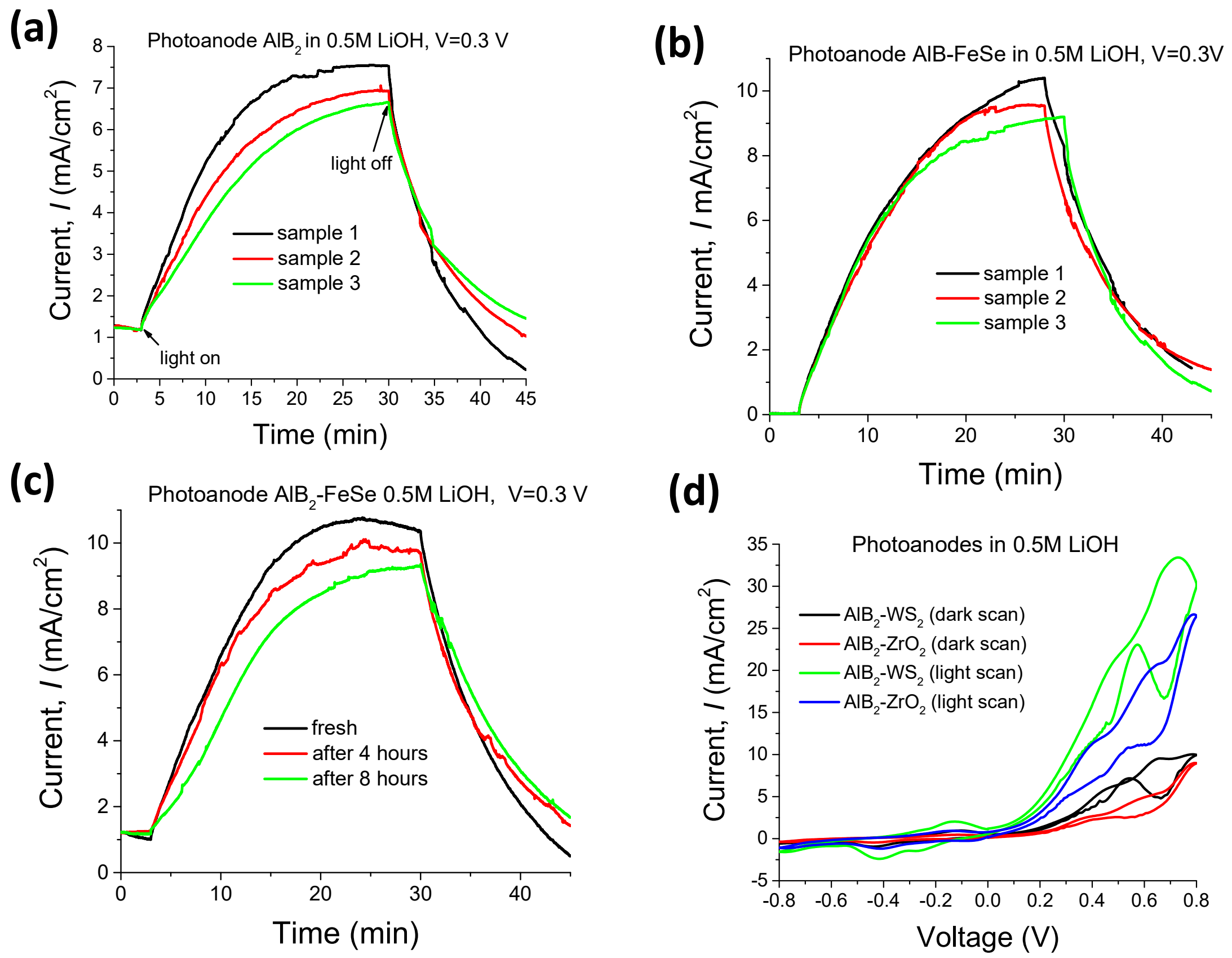

Figure S3. Reproducibility and stability of AlB_2 and AlB_2-FeSe photoanodes. Photocurrent as a function of time (V_{bias}=0.3 V), for the integrated water splitting device submerged in 0.5M LiOH electrolyte under illumination a solar light simulator: **(a)** three different AlB_2 photoanodes, **(b)** three different AlB_2-FeSe photoanodes. **(c)** Photocurrent as a function of time for AlB_2-FeSe photoanodes after immersion in 0.5M LiOH electrolyte for 0, 4 and 8 hours. **(d)** Cycling voltammetry scan of the investigated hybrid AlB_2-WS_2 and AlB_2-ZrO_2 nanostructured photoanodes in the 0.5M LiOH (compare dependences for the dark and light scans).

In Fig. S4 we compare Raman spectra of fresh samples and samples after the 8-hour water splitting in 0.5M LiOH electrolyte. Both spectra exhibit strong maxima corresponding main vibration modes resulted from vibrations Al-B (~563 and 965 cm^{-1}) and Fe-Se ~282 and 387

cm^{-1}) atoms and a group additional weak maxima for tested samples at ~417, 577 and 750 cm^{-1}). The energies of small peaks are typical for Raman investigations in Al_2O_3 crystals [S7] (lower energy correspondent to A_{1g} symmetry and higher energy to E_g ones)

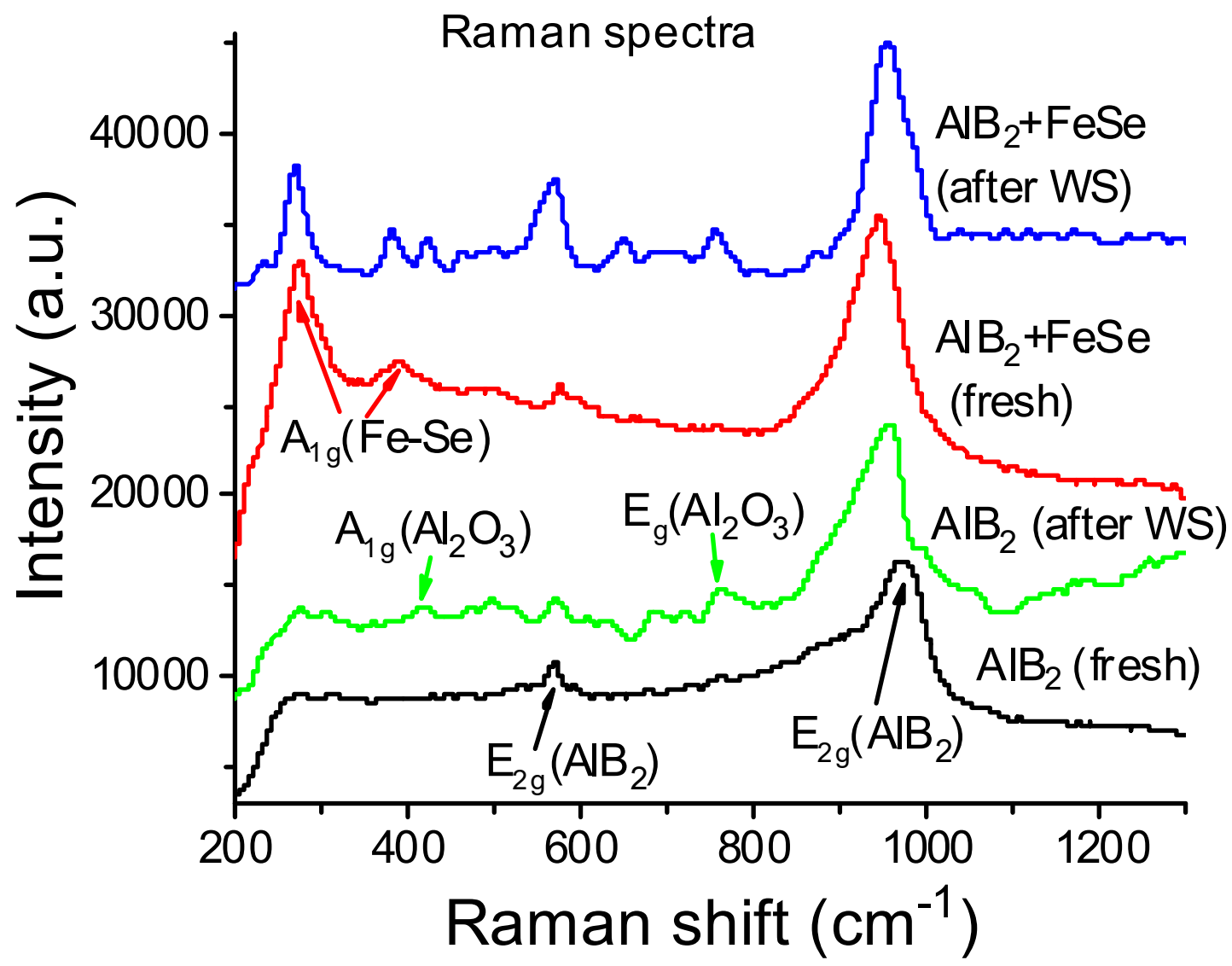

Figure S4. Evolution of Raman spectra for AlB_2 and AlB_2-FeSe nanostructures after photovoltaic water splitting during 8-hours stability test.

The applied bias photon-to-current efficiency (ABPE) was calculated using the standard equation [S8]

$$\mathrm{ABPE} = \frac{J_{SC}(\mathrm{mA\ cm^{-2}}) \times (1.23\mathrm{V} - V_{bias})}{P_{light}(\mathrm{mW\ cm^{-2}})}$$

where J_{SC} is short-circuit current density, V_{bias} is the applied potential between photoelectrode and counter electrode, P_{light} is the total irradiation input, and the redox potential of interest is equal to 1.23V for water oxidation.

The reproducibility of photocurrent values for AlB_2 and AlB_2-FeSe photo-anodes was estimated by repeated independent measurements for many studied samples. We found that different AlB_2 (AlB_2-FeSe) photoanodes exhibit similar ~5% variation of measured photocurrents (for AlB_2-FeSe photoanodes distribution is slightly larger ~7% than for plasmonic AlB_2 ones).

The monochromatic photon to current conversion efficiency (ABPE (λ)) can be estimated as [S9]

$$\mathrm{ABPE}(\lambda) = \frac{\mathrm{J_{SC}(mA\,cm^{-2})\times 1240}}{P_{\lambda}(\mathrm{mW\,cm^{-2})\times A\times \lambda(nm)}}$$

where λ is the single wavelength light source, J_{SC} (mA cm^{-2}) is the differential (light-dark) measured photocurrent for AlB_2 based nanostructured film, P_{λ} (mW cm^{-2}) is the measured power density of the light source at a specific wavelength, λ and A (cm^2) is the geometrical area of the illuminated electrode.

Raman spectroscopy. Surface-enhanced Raman scattering (SERS) is a physical phenomenon in which weak Raman signals of tested molecules are enhanced when they are close to the surface of plasmonic nanomaterials. In addition to the plasmonic effect, charge transport, and dipole formations, which are the main determining factors of SERS enhancement, some structural characteristics of 2D materials also play important roles in improving the Raman signals of detection molecules. We used the Raman spectroscopy to confirm of LSPR excitations and hence plasmonic mechanism in photocatalytic water splitting based on the hybrid AlB_2 NSs. For a Raman enhancement experiments, we used a 10^{-6} M of Rhodamine 6G (R6G) solvent in PMMA 950, 3 % anisole. This solution was then spin-coated onto the samples to form an approximately 100 nm thick film. The Raman measurements were performed using a Witec confocal scanning Raman microscope under a 514.5 nm laser light using a 1800 lines/mm grating with a x100 objective. The laser incident power did not exceed ~0.5 mW to avoid thermal damage or photo-bleaching of the R6G molecules and a laser spot size was of approximately 1 − 1.5 µm. Three characteristic peaks at ~1365, 1512, and 1615 cm^{-1} associated with R6G [S6] can be seen in Figure S5 for the cases of pure 10^{-6} M R6G on the Cu substrate, and a combination of hybrid AlB_2-2D NSs and 10^{-6} M R6G dye layer. We evaluated the SERS enhancement factor ($\eta = h_2/ h_1$) - the relative increase in the peak heights (intensities h_1 and h_2) from the photoanode samples compared with the plain substrate. We found that the resonant Raman signal of R6G dye is enhanced by ~20 times in magnitude by near-fields of plasmonic AlB_2 nanostructures, confirming the presence of LSPRs. Furthermore, it was shown that the SERS enhancement factor η decreases when the AlB_2 NSs are embedded into an ultrathin insulating layer ZrO_2, in which case only the near-field effect can be attributed to the photocatalytic reaction enhancement.

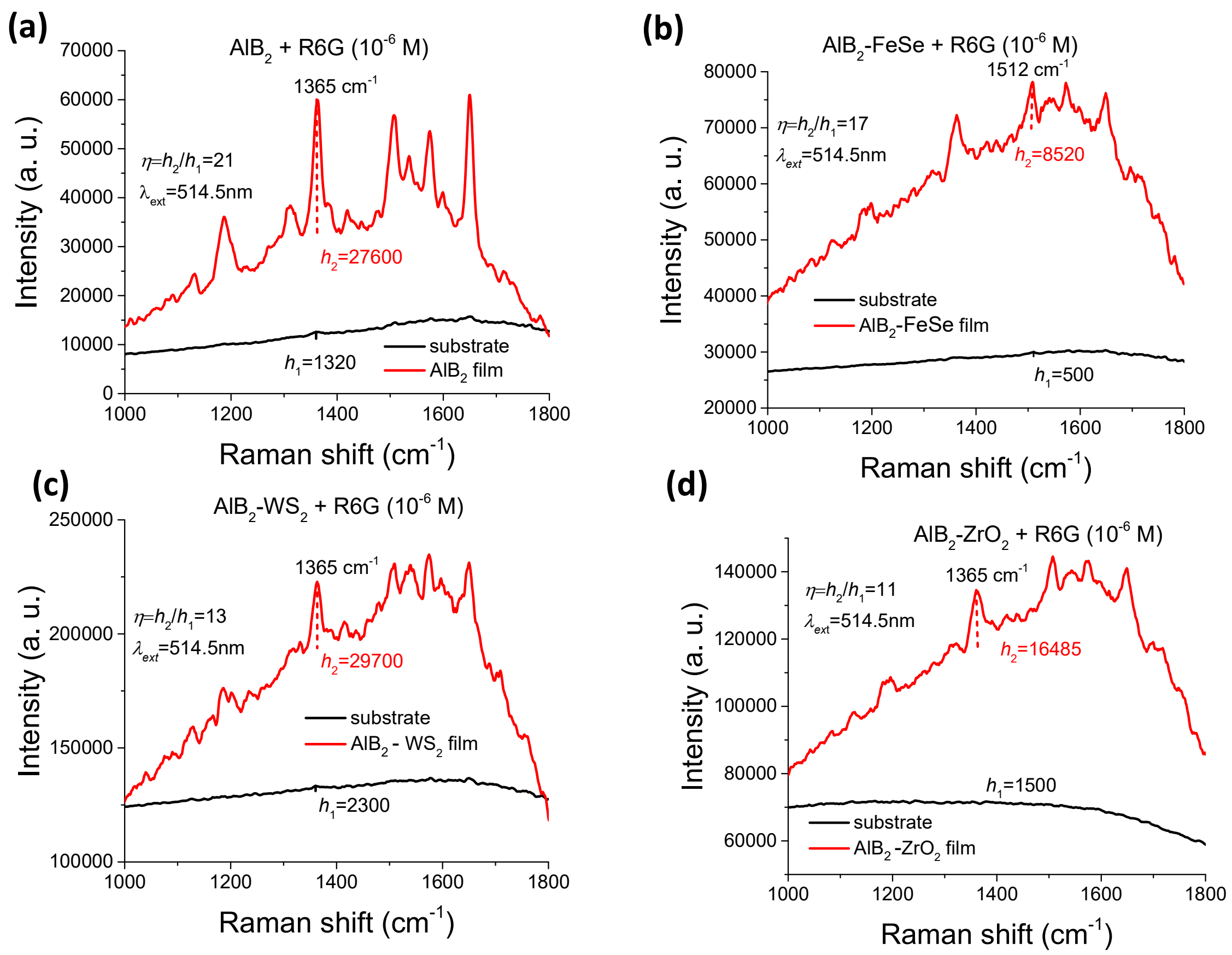

Figure S5. Surface enhanced Raman spectra from R6G dye molecules placed on the top of AlB_2-based 2D nanostructured photoanodes providing highly enhanced electromagnetic fields and a substrate measured with an excitation laser of λ=514.5 nm. **(a)** AlB_2 photo-anodes. **(b)** AlB_2-FeSe photo-anodes. **(c)** AlB_2-WS_2 photoanodes. **(d)** AlB_2-ZrO_2 photo-anodes.

Finite-difference time-domain (FDTD) simulations and effective-film optical modelling. Electromagnetic modelling was carried out to support the optical advantages of the AlB_2 based hybrid photoanode. The optical absorption and refection spectra of the electrodes were evaluated using a normal-incident transform-matrix model. The simulated stacks were composed of water/electrolyte (n=1.33) as the incident media, 250 nm effective films and the thick Cu substrates. The complex refractive indices of AlB_2, AlB_2-FeSe, AlB_2-WS_2 and AlB_2-ZrO_2 films were obtained from the ellipsometry measurements, and the optical constant of Cu was taken from the Palik database. The total reflectance was obtained as $R = |r|^2$, and the absorptance was $A = 1 - R$. The effective-film calculations show that all AlB_2-based electrodes have high optical absorption in the visible-NIR region, and the AlB_2-FeSe stack has the lowest broad band reflection among the investigated hybrid films. The mean total absorptance of AlB_2-FeSe reaches 0.957 over 400-1200 nm and 0.975 over 700-900 nm, which support the impedance-matching mechanism in the main text.

Table 2. Effective-film optical modelling results for AlB_2-based photoanode stacks.

Sample	Range (nm)	Mean R	Mean total absorptance A = 1 - R
AlB_2	400-1200	0.094	0.906
AlB_2-FeSe	400-1200	0.043	0.957
AlB_2-WS_2	400-1200	0.181	0.819
AlB_2-ZrO_2	400-1200	0.075	0.925
AlB_2	700-900	0.110	0.890
AlB_2-FeSe	700-900	0.025	0.975
AlB_2-WS_2	700-900	0.116	0.884
AlB_2-ZrO_2	700-900	0.092	0.908

Local field distributions were simulated by three-dimensional method in Ansys Lumerical FDTD. The simulation region was filled with a water/electrolyte background (n = 1.33), and perfectly matched layer (PML) boundary conditions were used to suppress artificial reflections. Disk-like geometries with diameter = 100 nm and thickness = 30 nm for AlB_2 and thickness = 20 nm for FeSe were used to simulate the flake material. The FeSe flake was placed above AlB_2 with a 2 nm gap and an 80 nm lateral distance, which simulates the partially overlapping AlB_2-FeSe interfaces in the rolled films. The optical constants of pure AlB_2 and FeSe were derived from ellipsometry experimental data. Normal incident plane waves were incident from the water side. A refined mesh of dx = 5 nm, dy = 5 nm and dz = 2 nm was used near the flake region. Two field monitors were placed at x-z cross-section trough the centre of the structure and x-y gap-plane, respectively. 500, 600, 700, 800 and 900 nm simulations were performed to evaluate the visible-NIR optical response of material. All field maps were normalized to a no-structure reference with identical source, monitors boundary conditions and water/electrolyte background, to remove the background field influence. The FDTD results show moderate but reproducible field enhancement near AlB_2 flake edges and near the AlB_2-FeSe interface. At 500 nm, the introduction of FeSe increases the maximum field factor F_{max} from 1.19 to 1.31 in the x-z section and from 1.01 to 1.26 in the x-y gap plane. At 900 nm, pure AlB_2 already shows stronger edge-localized fields, while AlB_2-FeSe still gives a modest additional enhancement. These calculations are consistent with the SERS evidence for plasmonic enhancement and the broad optical absorption implied by ellipsometry.

Table 3. Reference-normalized FDTD local-field enhancement factors for pure AlB_2 and AlB_2-FeSe disk-like nanoflake geometries. F_{max} is the maximum value of $F = |E\_structure|^2/|E\_reference|^2$ in the

monitor plane. P99 is the 99th percentile value, which is a more robust measure of the high-field region. The x-y maps were taken at the AlB_2-FeSe gap plane; For pure AlB_2, the same z-plane was used for comparison.

Wavelength (nm)	Monitor	Pure AlB_2 F_{max}	AlB_2-FeSe Fmax	Pure AlB_2 P99_F	AlB_2-FeSe P99_F
500	x-z	1.185452721	1.305328	1.047236831	1.145345962
500	x-y gap	1.00843999	1.261578	1.003725925	1.173083367
600	x-z	1.31485617	1.403562	1.107161122	1.220795774
600	x-y gap	1.078166232	1.348523	1.077991489	1.242008967
700	x-z	1.4865274	1.570945	1.200644807	1.312691194
700	x-y gap	1.189733324	1.408523	1.144669231	1.310560368
800	x-z	1.713531407	1.819922	1.34392011	1.409592892
800	x-y gap	1.38288585	1.503287	1.255981629	1.354988474
900	x-z	1.97202185	2.094093	1.543310763	1.542863786
900	x-y gap	1.670170036	1.78965	1.406739891	1.444415095

References

S1. Jiang, C.; Ma, Y.; Zhao, F.; Wei, L.; Zhang, H.; Pei, C. Synthesis and characterisation of AlB_2 nanopowders by solid state reaction. *Micro & Nano Letters* **2014**, *9* (2), 132–135. https://doi.org/10.1049/mnl.2013.0665.

S2. Wanga, Y.; Liana, J.; Weia, M.; Shic, Y.; Jinc, K.; Wang, C.; Zhanga, Y.; Xub, Z.; Zhoub, X.; Li, Y. Optical property and pseudogap study of FeSe thin films on different substrates. *Optical Materials* **2022,** *131*, 112727.

S3. Nabeshima, F.; Imai, Y.; Hanawa, M.; Tsukada, I.; Maeda, A. Enhancement of the superconducting transition temperature in FeSe epitaxial thin films by anisotropic compression. *Appl. Phys. Lett.* **2013**, *103,* 17. doi:10.1063/1.4826945.

S4. Nguyen, T. P.; Sohn, W.; Oh, J. H.; Jang H. W.; Kim, S. Y. Size-Dependent Properties of Two-Dimensional MoS_2 and WS_2. *The Journal of Physical Chemistry C*, **2016**, *120*, 10078-10085.

S5. Zare, M.H.; Mehrabani-Zeinabad, A. Photocatalytic activity of $ZrO_2/TiO_2/Fe_3O_4$ ternary nanocomposite for the degradation of naproxen: characterization and optimization using response surface methodology. *Scientific Reports* **2022**, *12*, 10388. https://doi.org/10.1038/s41598-022-14676-y

S6. Sil, S.; Kuhar, N.; Acharya, S.; Umapathy, S. Is Chemically Synthesized Graphene 'Really' a Unique Substrate for SERS and Fluorescence Quenching? *Scientific Reports* **2013**, *3* (1), 3336. DOI: 10.1038/srep03336.

S7. P. G. Li, M. Lei and W. H. Tang, "Raman and photoluminescence properties of α-Al2O3 microcones with hierarchical and repetitive superstructure," Materials Letters 64 (2), 161-163 (2010). https://doi.org/https://doi.org/10.1016/j.matlet.2009.10.032.

S8. S. Chu et al., "Roadmap on solar water splitting: current status and future prospects," Nano Futures 1 (2), 022001 (2017). https://doi.org/10.1088/2399-1984/aa88a1.

S9. V. G. Kravets and A. N. Grigorenko, "Water and seawater splitting with MgB2 plasmonic metal-based photocatalyst," Scientific Reports 15 (1), 1224 (2025). https://doi.org/10.1038/s41598-024-82494-5.
